# A Single Nucleotide Polymorphism Uncovers a Novel Function for the Transcription Factor Ace2 during *Candida albicans* Hyphal Development

**DOI:** 10.1371/journal.pgen.1005152

**Published:** 2015-04-15

**Authors:** Diana M. Calderón-Noreña, Alberto González-Novo, Sara Orellana-Muñoz, Pilar Gutiérrez-Escribano, Yolanda Arnáiz-Pita, Encarnación Dueñas-Santero, M. Belén Suárez, Marie-Elisabeth Bougnoux, Francisco del Rey, Gavin Sherlock, Christophe d’Enfert, Jaime Correa-Bordes, Carlos R. Vázquez de Aldana

**Affiliations:** 1 Instituto de Biología Funcional y Genómica, Consejo Superior de Investigaciones Científicas (CSIC)/Universidad de Salamanca (USAL), Salamanca, Spain; 2 Departamento de Ciencias Biomédicas, Facultad de Ciencias, Universidad de Extremadura, Badajoz, Spain; 3 Institut Pasteur, Unité Biologie et Pathogénicité Fongiques, Département Mycologie, Paris, France; 4 INRA, USC2019, Paris, France; 5 Department of Genetics, Stanford University, Stanford, California, United States of America; University College Dublin, Ireland

## Abstract

*Candida albicans* is a major invasive fungal pathogen in humans. An important virulence factor is its ability to switch between the yeast and hyphal forms, and these filamentous forms are important in tissue penetration and invasion. A common feature for filamentous growth is the ability to inhibit cell separation after cytokinesis, although it is poorly understood how this process is regulated developmentally. In *C*. *albicans*, the formation of filaments during hyphal growth requires changes in septin ring dynamics. In this work, we studied the functional relationship between septins and the transcription factor Ace2, which controls the expression of enzymes that catalyze septum degradation. We found that alternative translation initiation produces two Ace2 isoforms. While full-length Ace2, Ace2^L^, influences septin dynamics in a transcription-independent manner in hyphal cells but not in yeast cells, the use of methionine-55 as the initiation codon gives rise to Ace2^S^, which functions as the nuclear transcription factor required for the expression of cell separation genes. Genetic evidence indicates that Ace2^L^ influences the incorporation of the Sep7 septin to hyphal septin rings in order to avoid inappropriate activation of cell separation during filamentous growth. Interestingly, a natural single nucleotide polymorphism (SNP) present in the *C*. *albicans* WO-1 background and other *C*. *albicans* commensal and clinical isolates generates a stop codon in the ninth codon of Ace2^L^ that mimics the phenotype of cells lacking Ace2^L^. Finally, we report that Ace2^L^ and Ace2^S^ interact with the NDR kinase Cbk1 and that impairing activity of this kinase results in a defect in septin dynamics similar to that of hyphal cells lacking Ace2^L^. Together, our findings identify Ace2^L^ and the NDR kinase Cbk1 as new elements of the signaling system that modify septin ring dynamics in hyphae to allow cell-chain formation, a feature that appears to have evolved in specific *C*. *albicans* lineages.

## Author Summary

*Candida albicans* is a major fungal pathogen in immunologically compromised patients. A key virulence trait is its ability to switch between the yeast and hyphal forms. Whereas yeast cells are required for dissemination, the filamentous forms are important in tissue penetration and invasion. In order to make a hypha, cell separation must be inhibited after cytokinesis, although the full extent of its regulation remains unknown. Previously, we have shown that the inhibition of cell separation in hyphae requires a modification of the dynamic properties of septins, a conserved family of GTPases that normally form a ring at the site of cytokinesis. Here we describe new factors regulating septin dynamics during hyphal development. We have discovered that an alternative translation initiation of *ACE2* mRNAs gives rise to an Ace2 protein, Ace2^L^, with an extra 54 aa at the N-terminus that exhibits a localization and function different from Ace2 transcription factor. This Ace2^L^ protein is upregulated upon hyphal induction and regulates the incorporation of the Sep7 septin into the septin rings to avoid inappropriate activation of cell separation in hyphae. Finally, we present evidence suggesting that the NDR kinase Cbk1 interacts with Ace2^L^ to regulate this process.

## Introduction

*Candida albicans* is an opportunistic human pathogen that is able to grow in different morphological forms, including the unicellular budding yeast form, pseudohyphae or true hyphae in response to external cues (reviewed in [1,2]). A key difference between yeast and hyphae is that yeast cells activate cell separation immediately after cytokinesis to allow the mother and daughter cells to become two independent entities. On the contrary, during hyphal growth cell separation is inhibited to maintain the different hyphal compartments attached forming a long filament. Ace2 is the transcription factor that controls cell separation in both *S*. *cerevisiae* and *C*. *albicans* [3–5]. In *S*. *cerevisiae*, the RAM (for Regulation of Ace2 and Morphogenesis) network is a signalling pathway that controls cell separation and polarized growth (reviewed in [6]). The final effector of the RAM pathway is the complex formed by the NDR kinase Cbk1 and its coactivator Mob2, which localize to the cortical sites of growth during budding and to the daughter cell nucleus at the M-G1 transition [7–11]. One of the phenotypes of cells lacking any of these proteins is a defect in cell separation due to the loss of Ace2-dependent transcription [7,8]. Ace2 specifically localizes to the daughter nucleus in late M and G1, where it directs the expression of daughter-specific genes involved in the separation of daughter and mother cells, such as *ENG1*, *CTS1* and *SCW11* [8,11–13]. Cbk1 controls the daughter-specific nuclear accumulation of Ace2 by blocking its export from the daughter cell nucleus [14]. In *C*. *albicans*, cell separation is regulated by a similar mechanism to that of *S*. *cerevisiae* and it requires Ace2 and the components of the RAM pathway [4,15,16]. In addition, the Cbk1-Mob2 complex is also required for hyphal development, and the activity of this complex is regulated by the phosphorylation of Mob2 by Cdc28 [17]. In both *S*. *cerevisiae* and *C*. *albicans*, Ace2 localization to the daughter cell nucleus depends on the Cdc14 phosphatase [11,15].

Septins are GTP-binding proteins that assemble into hetero-oligomers and filaments and are important elements in morphogenesis in almost all eukaryotic organisms with the exception of plants [18–24]. In recent years, major advances have been made in our understanding of the structure and organization of septin filaments. Structural studies of human septins have revealed a non-polar arrangement of the filaments in which the septin subunits interact through two different interfaces, one involving the nucleotide-binding domain—termed the G interface—and another involving interactions between the N and C terminal ends, the NC interface [25]. Regardless of the organism, the organization of septin filaments is similar to the human complex. In *S*. *cerevisiae*, 5 septins are produced during vegetative growth, encoded by the *CDC3*, *CDC10*, *CDC11*, *CDC12* and *SHS1* genes [26,27]. In this organism, the basic polymerization unit is a hetero-tetramer, and two of these later join to form the nonpolar octamer Cdc11-Cdc12-Cdc3-Cdc10-Cdc10-Cdc3-Cdc12-Cdc11 [28]. Septins form a ring at the incipient bud site before bud emergence, and they are subsequently rearranged into an hourglass-shaped collar that spans the neck and persists until cytokinesis, when it splits into two rings [29,30]. Fluorescence Recovery After Photobleaching (FRAP) analysis of *S*. *cerevisiae* septin rings showed that during most of the cell cycle the septin rings are in a static or frozen state, where no lateral diffusion is observed. Only during bud emergence and cytokinesis are the septins found in a “fluid” state, in which the septins move around and rearrange themselves inside the ring.

*C*. *albicans* contains five septin orthologs: Cdc3, Cdc10, Cdc11, Cdc12 and Shs1 (known as Sep7). *CDC3* and *CDC12* are essential genes, while mutants lacking *CDC10* or *CDC11* are viable but show defects in cytokinesis [31,32]. The organization and dynamics of septin rings in yeasts is similar to that of *S*. *cerevisiae*, assembling a septin collar at the bud neck that splits in two at cytokinesis [33]. When yeast cells are induced to develop filaments, the septins initially assemble a diffuse structure known as the basal band, consisting of parallel bars. Later, the first hyphal septin ring is assembled inside the germ tube, which splits into two rings before cytokinesis. Septins can also be found as a faint cap at the tip of growing hyphae [31,32]. In hyphae, septin rings are maintained rather than disassembled after cytokinesis, resulting in multiple rings along the length of the hypha, each one marking a septation site. In addition, hyphal septin rings are converted to a “hyphae-specific” state (HSS) that is characterized by a highly dynamic state of Cdc10, which is constantly exchanged between the ring and a cytoplasmic pool [33]. This modification of septin ring dynamics is crucial for the inhibition of cell separation intrinsic to hyphal development, and it is dependent on Sep7 and on its phosphorylation by the hypha-specific cyclin-CDK complex Hgc1-Cdc28. *sep7*Δ mutants form normal hyphae upon induction, but the compartments separate after cytokinesis. In *C*. *albicans*, activation of the cell separation program after cytokinesis requires the Cdc14 phosphatase, which localizes to the septum of yeast cells during cytokinesis but not to the hyphal septum [15]. In *sep7*Δ mutants, Cdc14 can localize to the hyphal septum, which activates the Ace2-dependent cell separation program, and promotes hyphal cell separation by controlled degradation of the septum. This degradation occurs through the action of hydrolytic enzymes such as the chitinase Cht3 and the endo-glucanase Eng1 [34,35].

In this report, we identify new regulators of septin ring dynamics during hyphal growth. We show that alternative translation initiation of *ACE2* mRNAs produces a new Ace2 protein (Ace2^L^) with no transcriptional activity that is required for the conversion of septin rings to the HSS. Our results reveal that Ace2^L^ influences the incorporation of the Sep7 septin to hyphal septin rings to allow cell-chain formation. Interestingly, a natural single nucleotide polymorphism (SNP) that generates a stop codon in the ninth codon of Ace2^L^ is present in many *C*. *albicans* isolates and homozygosity for this polymorphism as seen in the *C*. *albicans* WO-1 lineage recapitulates the phenotype of cells lacking Ace2^L^. Finally, we report that impairing the activity of the NDR kinase Cbk1 results in a failure of conversion of septin rings to the HSS similar to that of hyphal cells lacking Ace2^L^.

## Results

### Ace2 influences septin dynamics in hyphae in a transcription-independent manner

**Fig 1:**
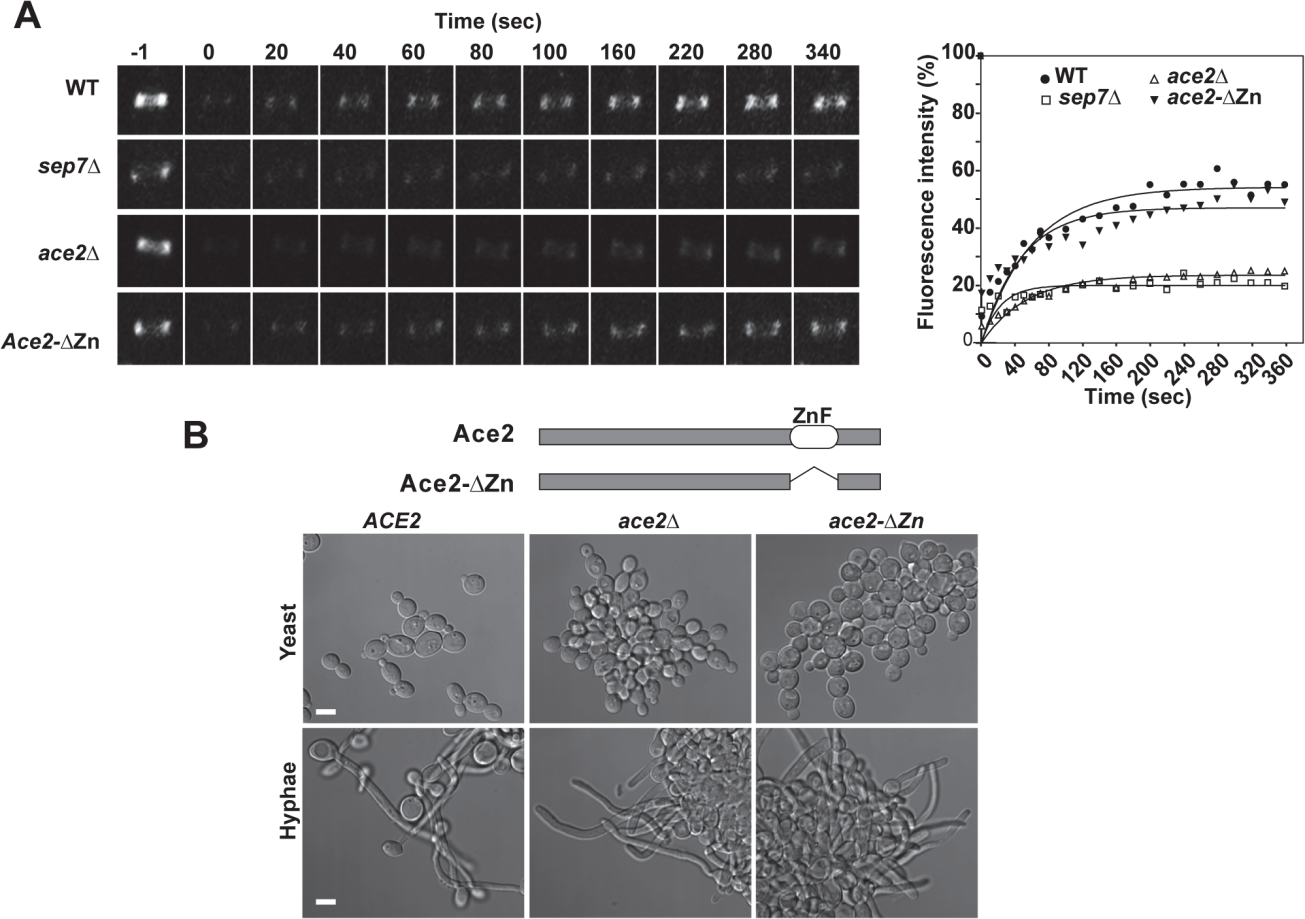
Ace2 controls septin ring dynamics during hyphal development. **A)** FRAP analysis of Cdc10-GFP dynamics in the wild type *CDC10-GFP* (*ACE2*, OL2244), *sep7*Δ/Δ *CDC10-GFP* (OL2182), *ace2*Δ/Δ *CDC10-GFP* (OL1444) and *ace2-*Δ*Zn/ace2*Δ *CDC10-GFP* (OL1551) strains. Rings were imaged using confocal spinning-disk microscopy. Individual rings were bleached and then imaged every 10 s for the first 80 s and then every 20 s. Images show the initial time (time -1) and the indicated times (seconds) after photobleaching. The graph represents the fluorescence intensity of the bleached regions corrected for cytoplasmic background and photobleaching. Statistical significance for the mobile fraction was determined by two-tailed t tests (Table 1). **B)** Schematic representation of Ace2 and Ace2-ΔZn. Ace2 contains two C2H2 zinc fingers close to the C-terminus that were deleted in Ace2-ΔZn. The images below show the phenotype of the wild-type strain (BWP17) and the *ace2*Δ/Δ (OL1444) and *ace2-*Δ*Zn/ace2*Δ (OL1551) mutants growing as yeasts or hyphae. Scale bar, 5 μm.

We have previously reported that septin dynamics is regulated developmentally to inhibit cell separation during the filamentous growth of *C*. *albicans* [33] in strain BWP17, which is a derivative of the reference *C*. *albicans* clinical isolate SC5314 [36]. Given that cell separation depends on the transcription factor Ace2, we decided to investigate the functional relationship between septins and Ace2 using the BWP17 background. As a first approach, septin dynamics was measured by FRAP experiments in wild-type and *ace2*Δ strains tagged with Cdc10-GFP. Entire septin rings were photobleached and fluorescence recovery was followed at 20-sec intervals (n≥14 for both strains). As previously described [33], wild-type hyphae showed a high Cdc10 turnover between the ring and the cytoplasm (mobile fraction around 40%) that was dependent on the Sep7 subunit (Fig 1A and Table 1). By contrast, *ace2*Δ/Δ hyphae exhibited a lower Cdc10 exchange (mobile fraction 22%), similar to that observed for *sep7*Δ/Δ mutants, suggesting a role for Ace2 in the regulation of Cdc10 dynamics during hyphal growth.

**Table 1:** Cdc10 FRAP summary statistics.

Strain	n	F end ± SD (%)	Mobile fraction ± SD (%)
WT	26	53.12 ± 6.61	43.26 ± 6.34
*sep7*Δ/Δ	19	38.13 ± 5.88	26.67 ± 4.66*
*ace2*Δ/Δ	18	31.19 ± 7.65	23.11 ± 3.71*
*ace2-*Δ*Zn*/*ace2*Δ	25	51.42 ± 7.12	43.18 ± 5.61
*ace2* ^*L*^/*ace2*Δ	24	46.91 ± 7.55	41.89 ± 6.81
*ace2* ^*S*^/*ace2*Δ	16	38.12 ± 5.40	27.2 ± 6.56*
*mob2-4A/mob2*Δ	18	35.94 ± 2.83	25.03 ± 2.53*

* P < 0.0001 (determined by two-tailed t tests)

Since Ace2 is a transcription factor [4,5], the most likely possibility seemed to be that an unknown Ace2-dependent gene would be necessary for Cdc10 exchange. To test this possibility, a heterozygous strain carrying a truncated version of *ACE2* in which the zinc fingers were deleted (*ace2-*Δ*Zn/ace2*Δ) was constructed. As expected, the *ace2-*Δ*Zn/ace2*Δ strain phenocopied the null *ace2*Δ/Δ strain (Fig 1B), suggesting that it lacked transcriptional activity. However, contrary to our expectation, FRAP experiments indicated that the kinetics of Cdc10 recovery in *ace2-*Δ*Zn/ace2*Δ hyphae was similar to that observed in the wild-type strain (Fig 1A and Table 1). Therefore, the Zn fingers of Ace2 are dispensable in hyphal cells suggesting that Ace2 influences septin dynamics independent of its transcriptional activity during hyphal growth.

### *ACE2* is transcribed in different mRNA forms

**Fig 2:**
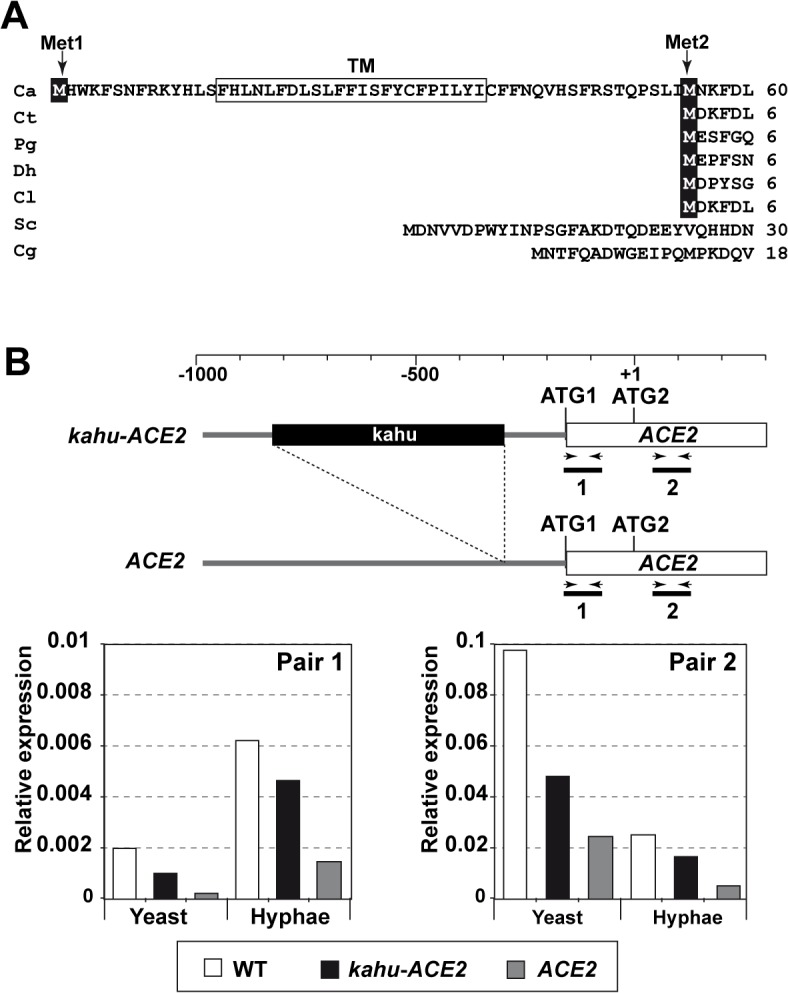
Different mRNAs and proteins are produced from the *ACE2* locus. **A)** Multiple sequence alignment generated by ClustalW of the N-terminus region of Ace2 from *C*. *albicans* (Ca), *C*. *tropicalis* (Ct), *Pichia guilliermondii* (Pg), *Debaryomyces hansenii* (Dh), *Clavispora lusitaniae* (Cl), *S*. *cerevisiae* (Sc) and *C*. *glabrata* (Cg). The two in-frame Mets are indicated (Met1 and Met2) and the predicted transmembrane region is boxed. **B)** qRT-PCR analysis of *ACE2* transcripts in the wild-type (BWP17), *ACE2*/*kahu-ace2*Δ (OL2037) and *kahu-ACE2*/*ace2*Δ (OL2035) strains. Schematic representation of the promoter region of the two *ACE2* alleles and the oligonucleotide pairs used for amplification. The graph represents the abundance of the two fragments in yeast or hyphae of the different strains normalized using *ADE2* expression.

The above results suggested that a domain present in the Ace2-ΔZn protein must be responsible for regulating septin dynamics. To study this further, Ace2 was analyzed using various databases (Pfam, SMART or InterPro). No conserved domains were identified, except the two known zinc fingers. Surprisingly, the SMART database indicated the presence of a putative transmembrane domain (TM, F15-I37) near the N-terminus. Interestingly, multiple sequence alignment of Ace2 from different yeasts species showed that this potential transmembrane domain was present in an additional 54-aa region that was absent in Ace2 orthologs from other yeasts (Fig 2A).

Since the sequence of *C*. *albicans* Ace2 contains a second in-phase methionine (M55) that aligns perfectly with the initial methionine of other species, and since this can be used as a translation initiation point to generate a protein without the putative TM region (Fig 2A), we first tested whether the coding region for the first 54 amino acids was present in the *ACE2* transcripts by quantitative real-time PCR (qRT-PCR) using mRNA isolated from yeast and hyphae. Two different pairs of primers were used (Fig 2B). The first pair was designed to amplify a DNA fragment of about 100 bp corresponding to the first ATG and the potential TM region (fragment 1, Fig 2B), while the second pair amplified a DNA region downstream of ATG2 (fragment 2). The quantitative results indicated that the fragment containing the first ATG and the potential TM region was present in a fraction of the *ACE2* transcripts in both yeast and hyphae (Fig 2B). In addition, the abundance of fragment 1 was upregulated upon hyphal induction, accounting for 24% of the total *ACE2* mRNAs present in hyphae. Thus, these results indicate the existence of different *ACE2* mRNAs species that change their relative amounts in response to the type of growth in *C*. *albicans*.

According to the *Candida* Genome Database (CGD [37]), the *ACE2* promoter is heterozygous and one of the alleles contains a long terminal repeat associated with the transposon Tca15 (known as *kahu-Ra* [38]) inserted 131 bp upstream of ATG1 which might affect its expression. To analyze whether the long mRNA is allele-specific, two strains carrying deletions of either the *ACE2* or the *kahu-ACE2* alleles were generated (*ace2*Δ/*kahu-ACE2* and *ACE2*/*kahu-ace2*Δ) and analyzed by qRT-PCR with the same primers. The results indicated that fragment 1 was present in both heterozygous strains, and its abundance increased upon hyphal induction as in the wild-type strain (Fig 2B). Therefore, the long mRNA is not allele-specific.

### Ace2^S^ functions as a transcription factor

**Fig 3:**
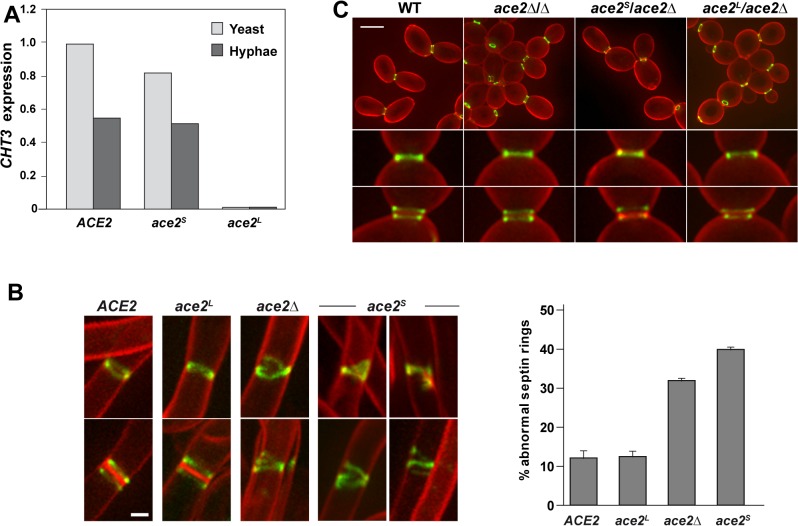
Phenotype of strains containing Ace2^S^ or Ace2^L^. **A**) Ace2^L^ lacks transcriptional activity. *CHT3* expression levels in the wild-type strain (BWP17) and in the *ace2* ^*S*^/*ace2*Δ (OL1598) and *ace2* ^*L*^/*ace2*Δ (OL1597) mutants analyzed by quantitative PCR. mRNA was obtained from yeast cells (light gray) or after the induction of filamentation (dark gray). The values obtained were normalized using the *ADE2* gene. **B**) Structure of septin rings during filamentation. Details of septin rings in strains *ACE2 SEP7-GFP* (CAG39), *ace2*Δ/Δ *SEP7-GFP* (OL1457), *ace2* ^*S*^ */ace2*Δ *SEP7-GFP* (OL1634) and *ace2* ^*L*^ */ace2*Δ *SEP7-GFP* (OL1631) during filamentation. The images show Sep7-GFP fluorescence (green) and calcofluor staining (red), and are the maximum projection of 10 planes acquired every 0.4 μm. Scale bar, 1 μm. The graph shows a quantification of the percentage of abnormal septin rings. The data are the mean of 2 experiments (n > 100 cells), and the standard deviation is indicated. **C**). Septin rings of the same strains during yeast growth. Cells were stained with calcofluor before visualization by fluorescence microscopy. The images are the maximum projection of 10 planes acquired every 0.4 μm and show Sep7-GFP fluorescence (green) and calcofluor staining (red). Below, details of single and duplicated rings of each strain. Scale bar, 5 μm.

Since transcription of the *ACE2* gene generated different mRNAs containing alternative translation initiation points, it is also possible that different isoforms of the Ace2 protein might be present in the cells. Translation initiation from ATG1 would generate a protein of 783 amino acids with an estimated molecular weight of 90.5 kDa containing the hypothetical TM region (henceforth termed Ace2^L^), while the use of ATG2 would result in a 729-amino acids protein (estimated molecular weight of 83.8 kDa) that could function as a transcription factor (henceforth termed Ace2^S^). To determine whether the two ATGs are used as translation initiation points, we generated strains expressing only one of the two proteins from its native promoter (*ace2* ^*S*^ */ace2*Δ and *ace2* ^*L*^ */ace2*Δ). In these strains, one of the two alleles was deleted while the other was replaced by a mutated version in which one of the two ATGs was changed to AAA by site-directed mutagenesis (S1A Fig). Thus, *ace2* ^*S*^ */ace2*Δ harbored a mutation in ATG1 expressing only the short form, whereas *ace2* ^*L*^ */ace2*Δ carried the mutation in ATG2, producing only Ace2^L^. Analysis of these cells during yeast growth showed that *ace2* ^*S*^ */ace2*Δ had a wild-type phenotype while *ace2* ^*L*^ */ace2*Δ cells phenocopied the null *ace2*Δ/Δ mutant (S1B Fig). Furthermore, the expression of the Ace2-target gene *CHT3* in *ace2* ^*S*^ */ace2*Δ cells was similar to that of the wild type whereas no expression was detected in the *ace2* ^*L*^ */ace2*Δ strain (Fig 3A). Thus, these results indicate that Ace2^S^ is the only form of Ace2 with transcriptional activity to induce the expression of genes involved in cell separation. During hyphal growth, Ace2-target genes are down-regulated by Efg1 [39]. The absence of Ace2^L^ did not disturb this regulation since *ace2* ^*S*^ */ace2*Δ cells were able to reduce the expression of *CHT3* to a level similar to that of the wild type control in response to hypha-inducing conditions (Fig 3A). This observation suggests that Ace2^L^ does not function as a transcriptional activator nor modify the activity of Ace2^S^ in hyphal cells.

### Ace2^L^ promotes normal septin ring assembly in hyphae

Given that Ace2^L^ lacked transcriptional activity and that Ace2 regulated septin dynamics in a transcription-independent manner (Fig 1A), we reasoned that Ace2^L^ could regulate septin ring dynamics in hyphae. To test this, we analyzed the septin organization in the *ace2* ^*L*^ */ace2*Δ and *ace2* ^*S*^ */ace2*Δ strains containing Sep7-GFP. Although both strains were able to develop hyphae in response to serum (S1C Fig), the structure of the septin rings was different. Hyphae from *ace2* ^*L*^ */ace2*Δ cells exhibited single and double septin rings similar to those observed in the wild-type (Fig 3B). By contrast, *ace2* ^*S*^ */ace2*Δ hyphae showed defects in septin organization, such as open rings, incorrectly compacted rings, V-shaped rings and even spirals in rare occasions, similar to those of the *ace2*Δ/Δ mutant (Fig 3B). Quantification of these defects indicated that *ace2* ^*S*^ */ace2*Δ and *ace2* null mutant hyphae presented a 3 to 4-fold increase of abnormal septin rings compared to the wild-type and *ace2* ^*L*^ */ace2*Δ hyphae. Similar results were obtained when Cdc10-GFP was analyzed, suggesting that these defects were not specific to Sep7 but probably affected the entire septin ring. Furthermore, this failure in septin ring formation was hypha-specific because no defects, either in single or double septin rings, were observed in yeast cells (Fig 3C). Therefore, these observations support the hypothesis that Ace2^L^ is important for normal septin ring assembly only during hyphal growth.

### Ace2^L^ is required to inhibit separation in hyphae

**Fig 4:**
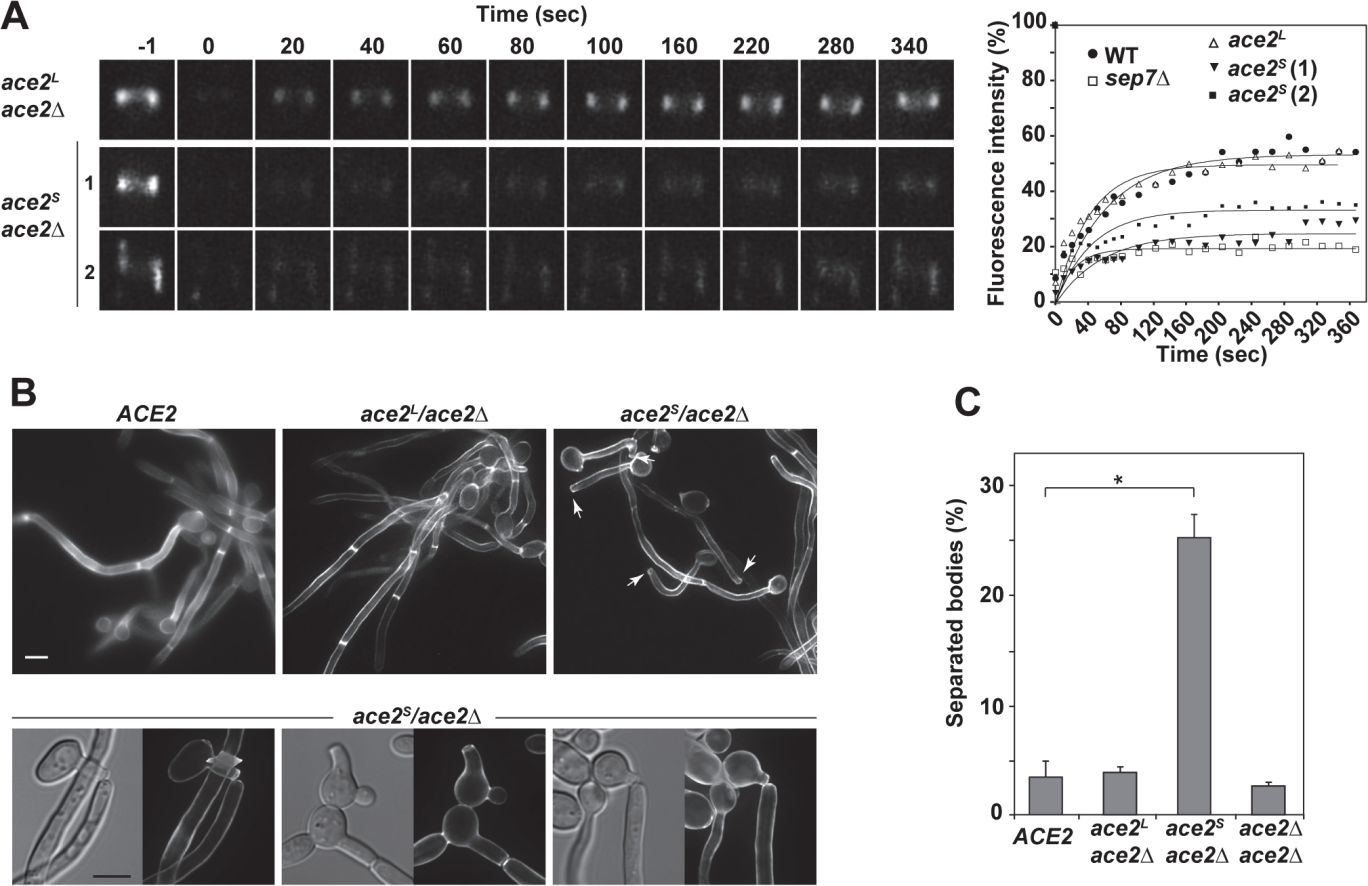
Ace2^L^ is essential for septin ring dynamics during hyphal growth. **A**) Cdc10 dynamics in strains *ace2* ^*S*^ */ace2*Δ *CDC10-GFP* (OL1659) and *ace2* ^*L*^ */ace2*Δ *CDC10-GFP* (OL1662). Complete rings were irradiated with the laser of a confocal microscope to remove the fluorescence and then imaged at the indicated times to analyze fluorescence recovery. The graph shows the quantification of the fluorescence in each ring. Statistical significance for the mobile fraction was determined by two-tailed t tests (Table 1). **B**) Fluorescence microscopy of calcofluor-stained filaments from the *ACE2* strain (BWP17) and the *ace2* ^*S*^ */ace2*Δ (OL1598) and *ace2* ^*L*^ */ace2*Δ (OL1597) mutants 3.5 h after the induction of filamentation. Arrows indicate separated bodies. Scale bar, 5 μm. Below, examples of separated bodies observed in strain *ace2* ^*S*^ */ace2*Δ. **C**) Quantification of the percentage of separated bodies observed in the wild-type and the *ace2* ^*S*^ */ace2*Δ, *ace2* ^*L*^ */ace2*Δ and *ace2*Δ*/ace2*Δ mutants. The data are means of 2 separate experiments (n > 150 cells), and the standard deviation is indicated. * P < 0.0001 (determined by two-tailed t tests).

Previously, we reported a hyphae-specific state (HSS) of the septin rings in *C*. *albicans*. A major feature of this HSS is the high turnover of Cdc10 between the septin ring and the cytoplasm [33]. Given that our data suggested a hypha-specific role of Ace2^L^ in septin ring assembly, we sought to determine whether Ace2^L^ was required for the conversion of septin rings to the HSS. To test this, we studied Cdc10 dynamics in septin rings of *ace2* ^*L*^ */ace2*Δ and *ace2* ^*S*^ */ace2*Δ hyphae by FRAP experiments. The results indicated that *ace2* ^*L*^ */ace2*Δ had similar dynamics to that of wild-type hyphae (mobile fraction around 44%), whereas *ace2* ^*S*^ */ace2*Δ was defective in conversion to HSS since the mobile fraction was reduced (around 26%), similar to that observed in the *ace2*Δ/Δ and *sep7*Δ/Δ mutants (Fig 4A and Table 1). Thus, Ace2^L^ is required for the conversion of septin rings to HSS in response to serum.

Modification of septin rings to the HSS is essential for cell separation to be inhibited during hyphal growth in *C*. *albicans* [33]. Given that *ace2* ^*S*^ */ace2*Δ was defective in conversion to HSS, an inappropriate activation of cell separation would be expected in these hyphae. Indeed, separated hyphal bodies in which the first septum was cleaved, isolated hyphae without a cell body or even hyphae in which the first septum was being degraded were frequently observed in the *ace2* ^*S*^ */ace2*Δ strain (Figs 4B and S1D). To confirm this observation, we quantified the percentage of separated cell bodies in cultures after 3 hours in the presence of serum at 37°C. Under these conditions, wild-type and *ace2* ^*L*^ */ace2*Δ cultures had less than 5% of separated cell bodies. By contrast, cell separation rose to 25% in *ace2* ^*S*^ */ace2*Δ hyphae (Fig 4C), suggesting that the absence of Ace2^L^ greatly increases the frequency of inappropriate cell separation in hyphae. These results therefore indicate that Ace2^L^ plays an important role in the control of septin ring dynamics that suppresses cell separation during hyphal growth.

### A single nucleotide polymorphism (SNP) in *ACE2* eliminates Ace2^L^ and increases hyphal separation in *C*. *albicans* WO-1 cells

To gain insight into the evolutionary origin of Ace2^L^, we compared the sequence of *ACE2* from species of the *Candida* clade. As mentioned above, the N-terminal extension of Ace2 was present in the *C*. *albicans* reference strain SC5314 [36]. We also analyzed the sequence of the WO-1 strain, characterized for white-opaque switching [40], which has recently diverged from SC5314 based on whole genome comparisons [41]. Interestingly, the WO-1 strain contains a homozygous SNP in the ninth codon of the *ACE2* ^*L*^ ORF (position 25, C to T) that introduces a stop codon that would not allow the translation of the long form of Ace2 (S2A Fig). Thus, WO-1 contains the TT genotype of this particular SNP, in contrast to BWP17 that has the CC genotype.

**Fig 5:**
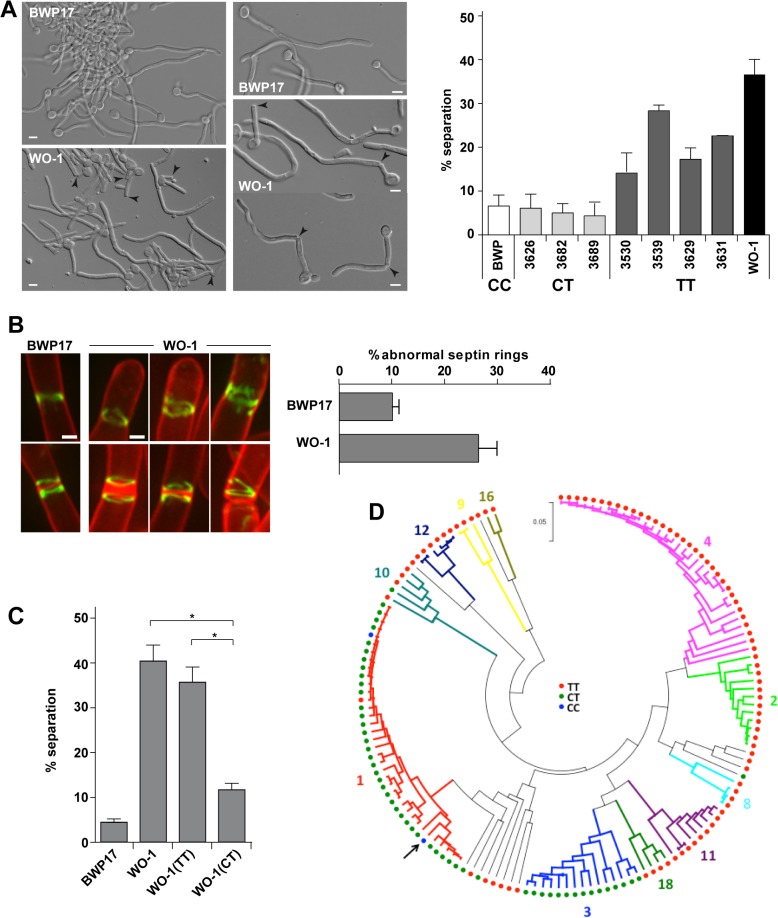
The WO-1 background contains a single nucleotide polymorphism (SNP) in *ACE2* that increases hyphal separation. **A**) Differential interference contrast (DIC) images from BWP17 and WO-1 hyphae (white cells) after 3.5 hours of induction of filamentation in the presence of serum at 37°C. General view and details of hyphae are shown. Arrowheads indicate separated bodies or hyphae undergoing separation. Scale bar, 5 μm. The graph represents the quantification of the percentage of separated bodies observed in BWP17 (white) and WO-1 (black) hyphae as well as in several natural *C*. *albicans* isolates from the CT (CEC3626, CEC3682 and CEC3689) or TT (CEC3530, CEC3539, CEC3629 and CEC3631) genotypes after 3.5 hours of filamentation. The data are means of 2 separate experiments (n >150 cells). **B**) Structure of septin rings during filamentation in the WO-1 background. Details of septin rings in BWP17 (OL2244) and WO-1 (WO-1 10G) hyphae carrying Cdc10-GFP. The images show Cdc10-GFP fluorescence (green) and calcofluor staining (red), and are the maximum projection of 15 planes acquired every 0.3 μm. Scale bar, 1 μm. The graph shows a quantification of the percentage of abnormal septin rings. The data are the mean of 2 experiments (n > 100 rings), and the standard deviation is indicated. **C)** Analysis of hyphal cell separation in strains BWP17, WO-1, WO-1(TT) and WO-1(CT). Cells were induced to filament in MM in the presence of serum at 37°C for 3.5 hours. The graph represents the quantification of the percentage of separated bodies observed in each strain. The data are means (±SD) of 3 separate experiments (n > 150 cells). * P < 0.002. **D)** Distribution of the genotypes at position 25 in the *ACE2* ^*L*^ ORF across *C*. *albicans* isolates. Genotypes were inferred from Illumina sequencing data obtained for 144 *C*. *albicans* commensal and clinical isolates from different geographical origins (MEB, GS and CD, manuscript in preparation). The phylogenetic relations between isolates based on their MLST type, their allocation to 11 of 18 clades (in different colors) structuring the *C*. *albicans* population and the genotypes are shown. The phylogeny was inferred in MEGA5 using the UPGMA method and the Maximum Composite Likelihood method for evolutionary distances. The arrow indicates the SC5314 strain.

If this SNP has created an additional form of Ace2 with a novel function in the SC5314 lineage but not in the WO-1 background, then it would be expected that WO-1 hyphae would have a rate of cell separation similar to that of BWP17 *ace2* ^*S*^ */ace2*Δ hyphae. Since white and opaque cells of the WO-1 strain have different environmental signals to induce the program of filamentous growth [42], WO-1 white cells were isolated by micromanipulation and induced to filament in the presence of serum at 37°C. After 3.5 hours of filamentation, separated bodies were observed in the WO-1 background (Fig 5A). Quantification of this phenotype indicated that cell separation in WO-1 hyphae was significantly higher than in BWP17 hyphae, and similar to that of *ace2* ^*S*^ */ace2*Δ hyphae. Furthermore, analysis of septin ring structures in WO-1 white hyphae using Cdc10-GFP showed the presence of abnormal septin structures similar to those found in BWP17 hyphae lacking Ace2^L^, such as open rings, incorrectly compacted rings or V-shaped rings (Fig 5B). Quantification of the percentage of these abnormal structures indicated that the frequency was higher than that of the wild-type BWP17 strain and similar to that found in BWP17 *ace2* ^*S*^ */ace2*Δ hyphae (Fig 5B). Therefore, WO-1 hyphae recapitulate the phenotypes observed in strains lacking Ace2^L^.

Since the rate of SNPs between the SC5314 and WO-1 strains is high (1 SNP per 160−195 bases) [41], it could be possible that this phenotype was due to other polymorphisms present in the genome. To confirm whether the mutation in *ACE2* was responsible for the cell separation phenotype, strain WO-1 (TT genotype) was converted to the CT genotype by mutating the TGA stop codon to CGA in one of the *ACE2* alleles and placing it under control of the *MET3* promoter (strain WO-1(CT)). As a control, we generated a strain carrying the *MET3* promoter upstream of the allele containing the TGA stop codon (strain WO-1(TT)). These strains were induced to filament in the presence of serum at 37°C and after 3.5 hours of filamentation, separated bodies were quantified (Fig 5C). The results indicated that strain WO-1(TT) had a percentage of separated hyphae similar to that of the parental WO-1 hyphae (35.68±6.01% versus 40.67±6.26%, respectively), whereas the percentage of separated bodies in the WO-1(CT) strain was significantly reduced (11.87±1.94%). Taken together, the results obtained from the WO-1 strain strongly support that Ace2^L^ has an important role in the inhibition of cell separation during hyphal growth in the SC5314 strain.

To gain further insights in the relevance of the SNP at position 25 in the *ACE2* ^*L*^ ORF distinguishing the SC5314 and WO-1 strains, we analyzed the genotypes of 144 *C*. *albicans* isolates from commensal or clinical origins and distributed across 11 of the 18 clades that structure the *C*. *albicans* population. Notably, a majority of the genotyped isolates (89 strains, 61.8%) harbored the TT genotype, similar to strain WO-1 (Fig 5D). 53 isolates (36.8%) almost exclusively from clades 1 and 3, were heterozygous (CT genotype) and only two clade 1 isolates (1.4%) including the SC5314 strain had the CC genotype. Genotypes showed strong association with clades (Fig 5D) suggesting that the CC genotype observed in the SC5314 strain had emerged recently. Since the presence of a single C allele (CT or CC genotypes) would result in the production of the Ace2^L^ form, it might be expected that hyphal separation would be lower in strains with either of these genotypes compared to those with the TT genotype. Indeed, when cell separation during hyphal growth was analysed in several of the natural isolates after 3.5 hours of hyphal induction, the percentage of separated bodies in CT strains was similar to that of the BWP17 strain (Fig 5A). In contrast, TT strains had higher values of cell separation, although they were variable between the different strains, perhaps indicating that other polymorphisms in the genome might affect the regulation of cell separation. Taken together, these results suggest a recent emergence of this *C*. *albicans*-specific Ace2 form in certain clades with a new function in the control of hyphal separation.

### Ace2^L^ is required for Sep7 incorporation to the septin ring during hyphal growth

**Fig 6:**
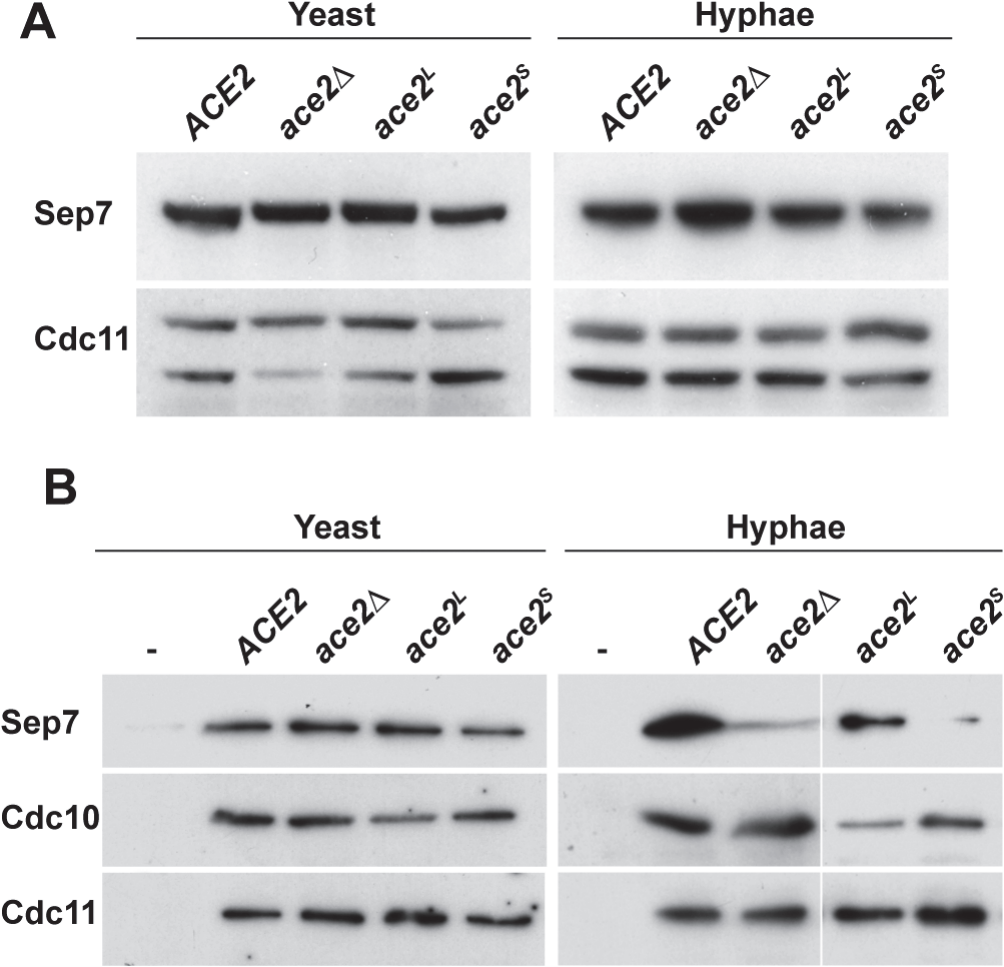
Absence of Ace2^L^ affects the incorporation of Sep7 to the septin rings. **A)** Western blot analysis from strains *ACE2 SEP7-HA* (BWP17 SHA), *ace2*Δ/Δ *SEP7-HA* (OL1453), *ace2* ^*L*^ */ace2*Δ *SEP7-HA* (OL1992) and *ace2* ^*S*^ */ace2*Δ *SEP7-HA* (OL1890) grown as yeast or hyphae. **B**) Immunoprecipitation of septin complexes. Septins were immunoprecipitated using RFP-TRAP during yeast or hyphal growth in strains *ACE2 CDC10-mCherry SEP7-GFP* (OL2086), *ace2*Δ/Δ *CDC10-mCherry SEP7-GFP* (OL2087), *ace2* ^*L*^ */ace2*Δ *CDC10-mCherry SEP7-GFP* (OL2088) and *ace2* ^*S*^ */ace2*Δ *CDC10-mCherry SEP7-GFP* (OL2090). Proteins in the immunoprecipitates were analyzed using anti-RFP, anti-GFP or anti-Cdc11 antibodies.

Our previous results have shown that the conversion of septin rings to HSS depends on the septin subunit Sep7, since hyphal cells depleted of Sep7 exhibit a low Cdc10 turnover and an inappropriate activation of cell separation ([33] and Fig 1A). Since *ace2* ^*S*^ */ace2*Δ hyphae phenocopied the *sep7*Δ/Δ mutant, we decided to study whether Sep7 regulation was disrupted in the absence of Ace2^L^. As a first approach, we wondered whether Sep7 protein levels might be dependent on Ace2^L^ in hyphal cells. To this end, we tagged *SEP7* with the HA epitope in the *ace2* ^*S*^ */ace2*Δ and *ace2* ^*L*^ */ace2*Δ strains. Western blot analysis of cell extracts from these cells indicated that Sep7 levels were not dependent on Ace2 function, because no significant changes in the amount of Sep7-HA were observed in the different *ace2* mutants, either in yeast or hyphae (Fig 6A).

We then analyzed the amount of Sep7 present in septin complexes by co-IP experiments using the *ACE2/ACE2*, *ace2*Δ/Δ, *ace2* ^*S*^ */ace2*Δ and *ace2* ^*L*^ */ace2*Δ strains carrying both the *CDC10-mCherry* and *SEP7-GFP* alleles at their chromosomal locus. Septin complexes were immunoprecipitated using anti-mCherry antibodies from yeast and hyphal extracts and the amount of Sep7, Cdc11 and Cdc10 in the precipitates was determined by Western Blot. Surprisingly, a significant reduction in Sep7 was observed in the Cdc10-IPs from the hyphae of strains lacking the Ace2^L^ form (*ace2*Δ/Δ and *ace2* ^*S*^ */ace2*Δ as compared to the wild-type and *ace2* ^*L*^ */ace2*Δ cells (Fig 6B). Interestingly, this reduction was Sep7-specific, since the amount of Cdc11 present in the septin complexes was similar in the four strains. Furthermore, this decrease was also hypha-specific because no differences were observed in Cdc10-IPs from yeast extracts (Fig 6B). Thus, Ace2^L^ is required for the incorporation of Sep7 to septin complexes during hyphal growth.

### Ace2 localizes to the nucleus and to cytoplasmic puncta

**Fig 7:**
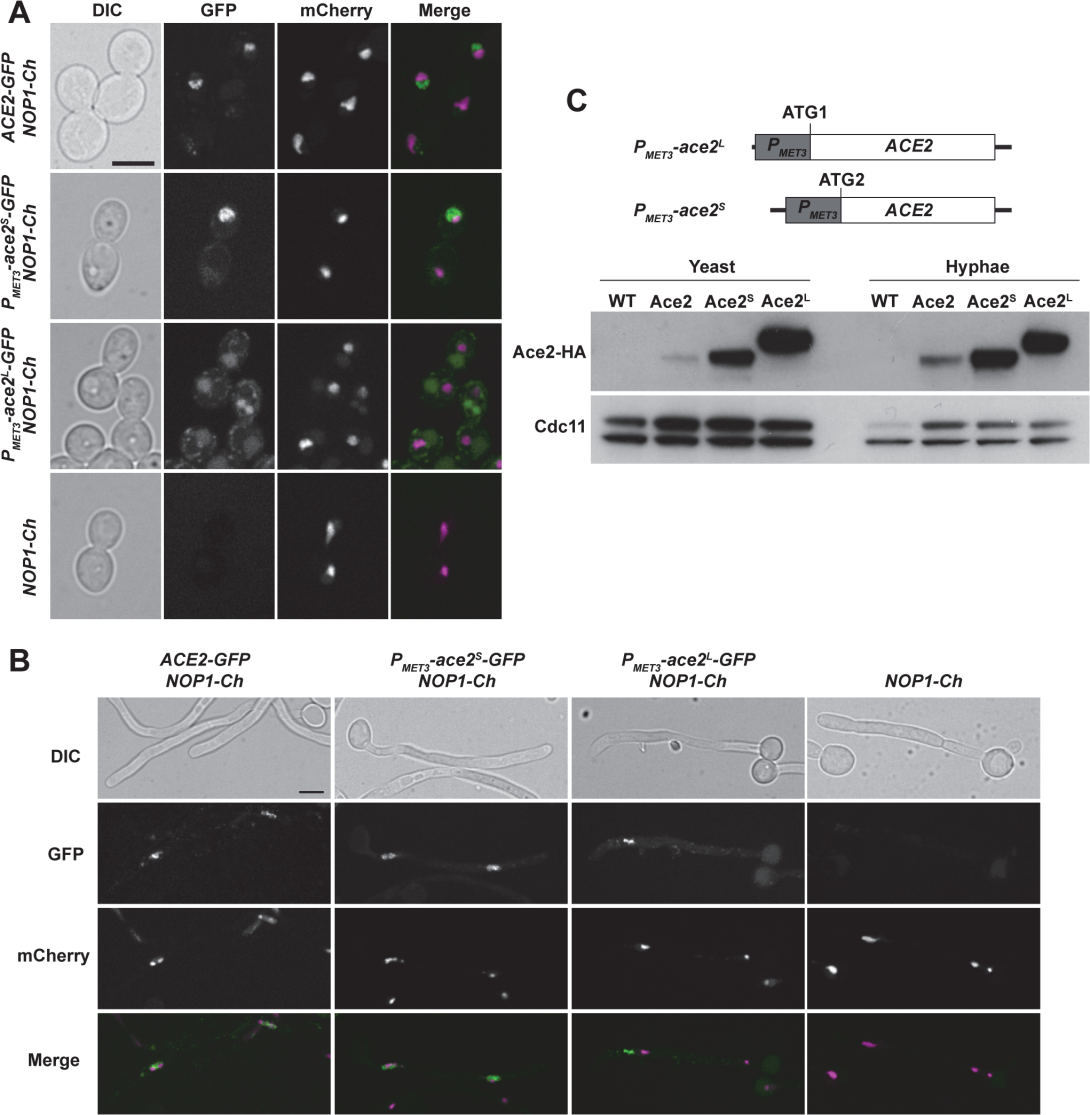
Localization of Ace2 in yeast and hyphae. **A**) Fluorescence microscopy of strains *ACE2-GFP NOP1-mCherry* (OL2345), *P*_*MET3*_ *-ace2* ^*S*^-*GFP NOP1-mCherry* (OL2356), *P*_*MET3*_ *-ace2* ^*L*^ *-GFP NOP1-mCherry* (OL2327) and *NOP1-mCherry* (OL2347) grown as yeasts. Individual DIC, Ace2-GFP and Nop1-mCherry channels are shown, together with the merged image of the GFP (green) and mCherry (magenta) signals. Bar, 5 μm. **B**) The same strains after induction of filamentation. **C**) Overexpression of Ace2^L^ and Ace2^S^. Schematic representation of the constructs used to overexpress Ace2^L^ and Ace2^S^ in which the *PMET3* promoter was cloned before ATG1 or ATG2, respectively. To the right, Western blot analysis of protein extracts from the untagged wild-type strain (WT, BWP17), *ACE2-HA* (Ace2, OL1538), *P*_*MET3*_ *-ace2* ^*L*^ *-HA* (Ace2^L^, OL1203) and *P*_*MET3*_ *-ace2* ^*S*^ *-HA* (Ace2^S^, OL1111) grown as yeast or hyphae. Anti-Cdc11 was used as loading control.

It has been shown that Ace2 accumulates in the nucleus in *C*. *albicans* [4]. However, our data so far suggested that the long isoform of Ace2 performs a function different to that of Ace2^S^, which is the form that acts as transcription factor. Thus, we decided to analyze the localization of Ace2 by fluorescence microscopy in strains carrying different forms of Ace2-GFP and Nop1-mCherry. Nop1 is an abundant nucleolar protein, and was used as marker for nucleus position [43]. The *ACE2-GFP NOP1-mCherry* strain expressed Ace2-GFP from its native promoter and the protein mainly localized adjacent to the nucleolar region in the daughter cell in yeast, as it has been described [4] (Fig 7A). Interestingly, during hyphal growth some cytoplasmic puncta were observed in addition to the nuclear signal (Fig 7B), suggesting that Ace2 expressed at physiological levels might have different localizations.

To differentiate between the localization of Ace2^S^ and Ace2^L^, we generated strains that expressed each of the two forms of Ace2 fused to GFP from the *MET3* promoter (*P*_*MET3*_ *-ace2* ^*S*^ *-GFP* and *P*_*MET3*_ *-ace2* ^*L*^ *-GFP*) and contained *NOP1-mCherry*. The fluorescence of Ace2^S^-GFP was present in the nucleus in both types of growth (Fig 7A and 7B). By contrast, Ace2^L^-GFP cells did not exhibit nuclear localization and the fluorescence was only found concentrated in cytoplasmic puncta (Fig 7A and 7B). These puncta did not correspond to a non-specific signal since they were absent in the *NOP1-mCherry* control strain. Therefore, these results indicate that the two forms of Ace2 localize to different subcellular compartments, Ace2^S^ being predominantly a nuclear protein while Ace2^L^ localizes as cytoplasmic puncta.

### Two different forms of Ace2

To test whether the two Ace2 isoforms were present in *C*. *albicans*, *ACE2* was tagged with the HA epitope at the C-terminus. Protein extracts were prepared from yeast or hyphae and analyzed by Western blot assays. The results disclosed the presence of a single diffuse band in both yeast and hyphae (Fig 7C). To confirm that Ace2^L^ is a stable protein, we constructed strains in which only one of the two forms was expressed under the control of the *MET3* promoter (*P*_*MET3*_-*ace2* ^*L*^ and *P*_*MET3*_-*ace2* ^*S*^). The *P*_*MET3*_-*ace2* ^*S*^ strain contained the *MET3* promoter [44] cloned upstream of ATG2, whereas strain *P*_*MET3*_-*ace2* ^*L*^ harbored the promoter upstream of ATG1 of an *ACE2* ORF in which the second ATG was destroyed by site-directed mutagenesis (Fig 7C). The analysis of cell extracts from yeast or hyphal cells in these two strains indicated that both Ace2 isoforms can be produced in *C*. *albicans* (Fig 7C).

### Domain analysis of Ace2^L^ function during hyphal growth

**Fig 8:**
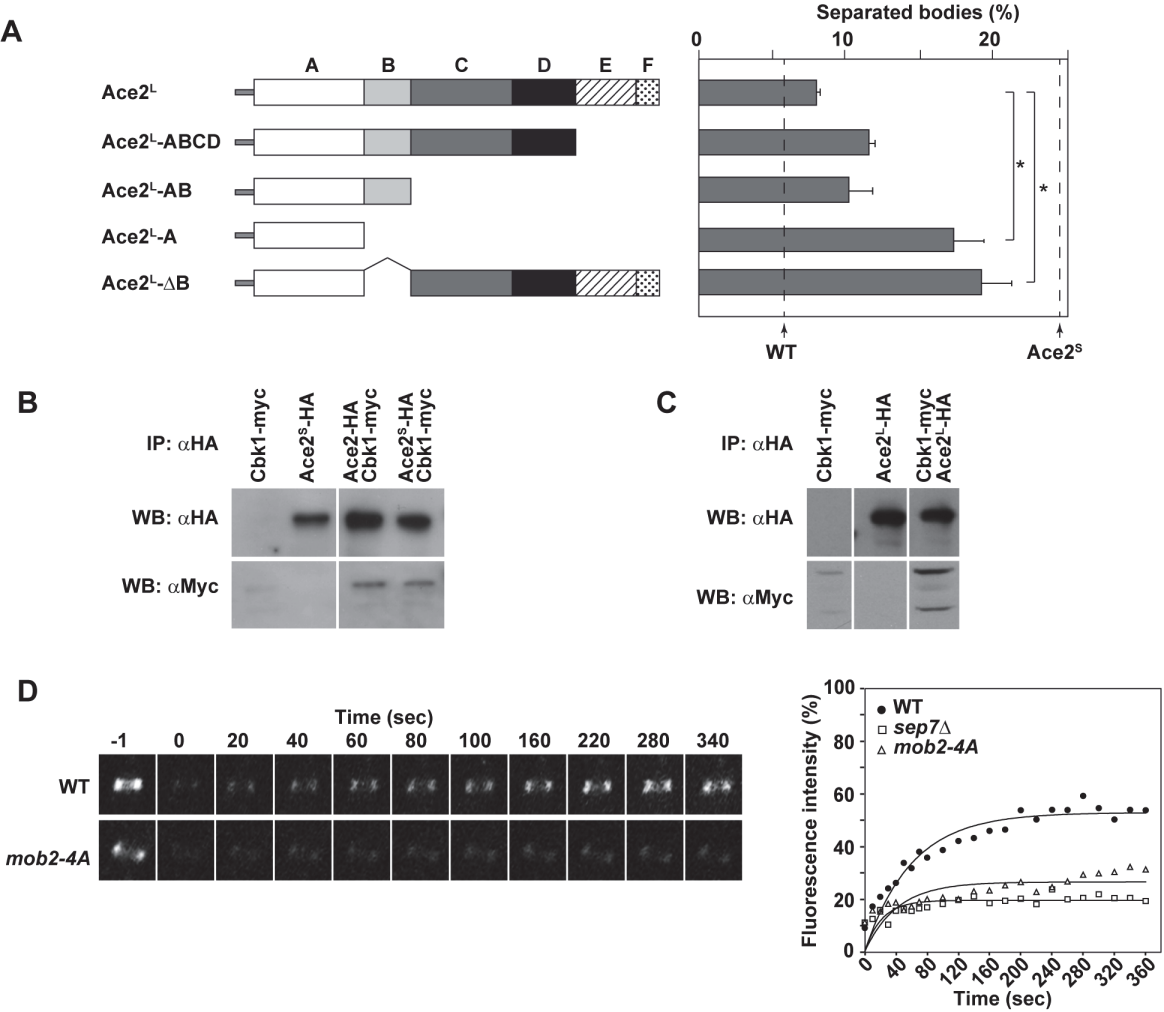
Analysis of Ace2 regions required to inhibit cell separation in hyphae. **A**) Schematic representation of the truncated versions of Ace2^L^ generated to analyze cell separation in hyphae, which were inserted into the *RPS1* locus under the control of the *MET3* promoter. The graph on the right represents the quantification of separated bodies observed in the filaments of strains *ace2* ^*S*^ */ace2*Δ *ace2* ^*L*^::*RPS1* (OL1170), *ace2* ^*S*^ */ace2*Δ *ace2* ^*L*^ *-ABCD*::*RPS1* (OL1874), *ace2* ^*S*^ */ace2*Δ *ace2* ^*L*^ *-AB*::*RPS1* (OL1716), *ace2* ^*S*^ */ace2*Δ *ace2* ^*L*^ *-A*::*RPS1* (OL1714) and *ace2* ^*S*^ */ace2*Δ *ace2* ^*L*^-Δ*B*::*RP10* (OL1876). The percentages of separation observed in the wild-type (BWP17) and the *ace2* ^*S*^ */ace2*Δ mutant (OL1598) are indicated with dashed lines. The data are means of 2 separate experiments (n > 150 cells), and standard deviations are indicated. *P < 0.0001 (determined by two-tailed t tests). **B**) Ace2 interacts with the Cbk1-Mob2 complex. Protein extracts from strains *CBK1-myc* (JC305), *P*_*MET3*_ *-ace2* ^*S*^ *-HA* (OL1111), *ACE2-HA CBK1-myc* (OL1719) and *P*_*MET3*_ *-ace2* ^*S*^ *-HA CBK1-myc* (OL1723) grown as yeast were immunoprecipitated using anti-HA antibodies. Samples were separated by SDS-PAGE and analyzed with anti-HA or anti-myc antibodies. **C**) Ace2^L^ interacts with the Cbk1-Mob2 complex. Protein extracts from strains *CBK1-myc* (OL1519), *P*_*MET3*_ *-ace2* ^*L*^ *-HA* (OL1203) and *P*_*MET3*_ *-ace2* ^*L*^ *-HA CBK1-myc* (OL1994) grown as yeast were immunoprecipitated using anti-HA antibodies. Samples were separated by SDS-PAGE and analyzed with anti-HA or anti-myc antibodies. **D)** Cdc10 dynamics in strains *CDC10-GFP* (OL2244) and *mob2-4A/mob2*Δ *CDC10-GFP* (JC1165). Complete rings were irradiated with the laser of a confocal microscope to remove the fluorescence and then imaged at the indicated times to analyze fluorescence recovery. The graph shows the quantification of the fluorescence in each ring. Statistical significance for the mobile fraction was determined by two-tailed t tests (Table 1).

In *S*. *cerevisiae*, six regions or subdomains have been described in the transcription factor Ace2, all performing different functions [45,46] (Fig 8A). Region A (amino acids 1–200) contains nuclear localization and nuclear export (NES) signals and two Cbk1-Mob2 phosphorylation sites; region B (201–301) is necessary for interaction with Cbk1-Mob2, and together with region A is important for Ace2 localization to the daughter cell nucleus. Region C (302–469) is required for the transcriptional activation of targets, while region D (470–577) has no known function and contains multiple CDK phosphorylation sites. Finally, regions E (578–692) and F (693–770) harbor the zinc fingers and another nuclear localization signal, respectively. To analyze whether this modular structure was conserved in *C*. *albicans*, the sequence of Ace2 proteins from *S*. *cerevisiae* and *C*. *albicans* were aligned. Although the overall similarity was low (24.5% identity and 34.6% similarity), some regions were conserved in both proteins, such as regions A (31.1% similarity), B (47.3%) and E (60.8%). By contrast, regions C, D and F were highly divergent, with similarity percentages below 25% (S2B Fig).

To further characterize Ace2^L^, we analyzed the regions of Ace2^L^ required for its function during hyphal growth. Strains carrying different regions of the coding sequence of *ACE2-HA* (from ATG1) under the control of the *MET3* promoter were integrated at the *RPS1* locus in the *ace2* ^*S*^ */ace2*Δ background. Western blot analysis showed that the different truncated proteins were expressed (S2C Fig). When cell separation in hyphae was analyzed, the results showed that regions C to F were dispensable to complement the phenotype of the *ace2* ^*S*^ */ace2*Δ mutant, since the percentage of cell separation in the strains harboring deletions of these regions was similar to that of the control *ace2* ^*S*^ */ace2*Δ *ace2* ^*L*^::*RPS1* (Fig 8A). By contrast, the strain expressing only domain A (Ace2^L^-A) was unable to complement the phenotype, suggesting that region B was required for Ace2^L^ function. To confirm this observation, we made a truncated version of Ace2^L^ where only region B was deleted (Ace2^L^-ΔB). Interestingly, hyphal cells expressing Ace2^L^-ΔB phenocopied the Ace2^L^-A mutant, confirming that region B is required for Ace2^L^ function during hyphal growth.

### Ace2^S^ and Ace2^L^ interact with the NDR kinase Cbk1 *in vivo*

In *S*. *cerevisiae*, region B is required for the interaction between Ace2 and the NDR kinase Cbk1 [47]. To check whether both Ace2 forms were able to interact with this kinase, co-immunoprecipitation assays (co-IPs) were conducted. To this end, Cbk1 was tagged with the c-myc epitope at the C-terminus in the *ACE2-HA*, *P*_*MET3*_ *-ace2* ^*S*^ *-HA* and *P*_*MET3*_ *-ace2* ^*L*^ *-HA* strains. Cell extracts from yeast cells were immunoprecipitated with anti-HA antibodies and analyzed by Western blot with anti-HA or anti-myc. The results of these co-IP experiments indicated that Cbk1 was present in all immunoprecipitates (Fig 8B and 8C), indicating that both Ace2 forms are able to bind to the NDR kinase Cbk1.

### Hyphae-specific septin dynamics require normal Cbk1 function

The physical interaction between Ace2^L^ and Cbk1 and the observation that region B was necessary for Ace2^L^ function suggested that this kinase would also be required for the conversion of the septin rings to the HSS. Since *cbk1*Δ/Δ mutants are defective in polarized growth and are unable to form hyphae [16], we used a hypomorphic mutant of the Cbk1-regulatory subunit Mob2 for FRAP experiments. The *mob2-4A* mutant is able to form short hyphae with enlarged tips that display an inappropriate activation of cell separation [17]. FRAP experiments in a *mob2-4A CDC10-GFP* strain indicated that the septin rings were deficient in Cdc10 exchange, since the mobile fraction was around 26% (Fig 8D and Table 1), similar to the values observed for *ace2*Δ/Δ and *sep7*Δ/Δ mutants. Therefore, these results suggest that the Cbk1-Mob2 complex is involved in alteration of septin dynamics during *C*. *albicans* hyphal growth.

## Discussion

In *S*. *cerevisiae* and other yeasts, cell separation depends on the transcription factor Ace2 [3,12,48]. This well conserved function of Ace2 requires its transcriptional activity to induce the expression of the genes encoding the cell wall hydrolases involved in septum dissolution [4,5,8,13,48–50]. In this report, we have characterized a new form of Ace2 in *C*. *albicans*, termed Ace2^L^, which influences septin dynamics and cell separation in a transcription-independent manner during hyphal growth.

Our results provide several key pieces of evidence that support the existence of Ace2^L^ with a different function than Ace2^S^. First, analysis of the *CaACE2* sequence from the CGD suggested that this gene is able to encode two different proteins by alternative translation initiation points (Fig 2A). The use of the first ATG would produce a protein with an extra 54-aa fragment at the N-terminus that harbors a putative TM domain (Ace2^L^), whereas the use of the second ATG would give rise to a protein with a N-terminal similar to that of Ace2 orthologs from other yeasts that act as transcription factors (Ace2^S^). Second, by using qRT-PCR we were able to detect different mRNAs with the genetic information to encode both proteins (Fig 2B). Furthermore, the abundance of the long mRNA was regulated developmentally since it increased 3-fold upon hyphal induction. Third, the *ace2* ^*S*^ */ace2*Δ and *ace2* ^*L*^ */ace2*Δ strains, which each expressed only one of the two Ace2 isoforms at physiological levels, produced distinct phenotypes (Figs S1B, 3 and 4). This is strong genetic evidence in support of the existence of two different Ace2 forms with unrelated functions. Finally, we have shown that hyphae from WO-1 white cells have a rate of cell separation similar to the *ace2* ^*S*^ mutant (Fig 5). This phenotype is largely due to a homozygous SNP that introduces a stop codon (TT genotype) that prevents the synthesis of Ace2^L^. Indeed, conversion of one of the alleles of this SNP to the nucleotide present in the SC5314/BWP17 background (CT genotype) highly reduced the separation of WO-1 hyphal bodies. Furthermore, analysis of hyphal separation of several *C*. *albicans* natural isolates showed that strains predicted to produce Ace2^L^ (CT or TT genotypes) had a reduced frequency of separated bodies, whereas strains of the TT genotype (that should not produce Ace2^L^) had higher rates of separated hyphae. Interestingly, there was a variation in the percentage of separated bodies observed in strains belonging to the TT genotype, perhaps reflecting the natural variability of these isolates.

The different phenotypes of strains *ace2* ^*S*^ */ace2*Δ and *ace2* ^*L*^ */ace2*Δ during yeast growth clearly indicated that the transcriptional activity was associated with Ace2^S^, since *ace2* ^*S*^ */ace2*Δ yeast cells separated similar to the wild-type, whereas *ace2* ^*L*^ */ace2*Δ yeast cells mimicked the phenotype of the *ace2*Δ/Δ mutant. Furthermore, the expression of the Ace2-target gene *CHT3* in *ace2* ^*S*^ */ace2*Δ cells was similar to that of the wild type whereas no evidence of transcriptional activity associated with Ace2^L^ was found (Fig 3A). Together, these findings clearly indicate that the transcriptional activation of the genes involved in cell separation lies in Ace2^S^.

If Ace2^L^ does not act as a transcription factor, what is its function? Our results indicate that Ace2^L^ plays a role in the modification of septin rings upon hypha induction (Figs 3 and 4). The dynamics of Cdc10-GFP in cells depleted of Ace2^L^ (*ace2*Δ/Δ or *ace2* ^*S*^ */ace2*Δ) suggests a failure in the conversion of septin rings to the HSS. This observation is in agreement with the inappropriate activation of cell separation observed in *ace2* ^*S*^ */ace2*Δ hyphae, since the HSS of septin rings is essential to inhibit cell separation during hyphal growth [33]. The cell separation phenotype of *ace2* ^*S*^ */ace2*Δ hyphae is not due to an upregulation of Ace2-target genes since Ace2^L^ did not modify the activity of Ace2^S^ in hyphal cells (Fig 3A). For cell separation to occur, polarized exocytosis of cell wall hydrolases to the septum region is required. In yeast, septins play a role in guiding exocytocis [29,51]. In *C*. *albicans*, it has been suggested that hyphal septin rings are modified by an unknown mechanism to restrict their ability to attract exocytocis to the division plate [51,52]. We postulate that the failure to convert the septin ring into the HSS of *ace2* ^*S*^ */ace2*Δ hyphae allows the targeting of vesicles carrying cell wall hydrolases to the septum.

One question that remains unsolved is what the molecular mechanism by which Ace2^L^ controls septin dynamics might be. The conversion of septin rings to the HSS depends on Sep7 [33]. Our data suggest a role for Ace2^L^ in the regulation of Sep7 affinity for septin complexes in hyphae (Fig 6B). The Ace2^L^-dependent regulation of Sep7 is probably indirect, since no physical interaction between Ace2^L^ and Sep7 has been detected in co-IP experiments. Little is known about how cytoplasmic septin subunits are incorporated into high-order structures such as rings. In *S*. *cerevisiae*, it has been reported that when Shs1 replaces Cdc11 at the ends of the octamers they do not assemble into filaments but associate laterally, forming curved rings [53]. Since the HSS in *C*. *albicans* depends on the Sep7/Shs1 subunit, it is possible that the proportions of Sep7 and Cdc11 monomers incorporated at the ends of the octamers during yeast or hyphal growth could be different, resulting in different higher-order structures. Therefore, the reduction in the amount of Sep7 observed in the *ace2*Δ/Δ and *ace2*Δ/*ace2* ^*S*^ mutants could modify the properties of the rings. Based on our results, we speculate that Ace2^L^ dots could act as docking sites for other hypha-specific factors involved in Sep7 regulation.

An interesting observation is that domain B of Ace2^L^ is required for its function (Fig 8A). In *S*. *cerevisiae*, this region drives the interaction between Ace2 and Cbk1, which is required for the asymmetric localization of Ace2 in the daughter nucleus at the M/G1 transition [8,12,46]. In *C*. *albicans*, Cbk1 also physically interacted with Ace2^S^ and Ace2^L^ (Fig 8B and 8C). Since domain B is well conserved between *Sc*Ace2 and *Ca*Ace2 (47.3% similarity), it is likely that it could be also required for such an interaction in *C*. *albicans*. Thus, the requirement of domain B for Ace2^L^ function suggests that the interaction with Cbk1 would be necessary to regulate septin dynamics in hyphae. Indeed, this seems to be the case, since the hypomorphic *mob2-4A* mutant was also defective in the conversion of the septin rings to the HSS (Fig 8D). Recently, it has been shown that Mob2 is phosphorylated by Cdks upon hyphal induction to inhibit cell separation [17]. It is possible that the interaction between Ace2^L^ and the hypha-specific isoforms of the Cbk1/Mob2 complex would be required to phosphorylate certain specific substrates involved in septin regulation. Future work will address the relevance of putative Cbk1 phosphorylation sites in possible targets involved in the regulation of septin dynamics.

*C*. *albicans* is a heterozygous diploid with a high degree of genetic variability among isolates [54,55]. Our analysis of a collection of 144 genetically-diverse *C*. *albicans* isolates showed that the genotypes giving rise to the Ace2^L^ isoform (TC and CC) are almost specific to clade 1 and 3 whereas isolates from the other clades have a genotype (TT) that only allows for the production of Ace2^S^. Clades 1 and 3 are among the five predominant clades in the *C*. *albicans* population and it has been proposed that clade 1 isolates are better adapted to colonize and invade epithelial surfaces [54,56,57]. Interestingly, isolates with the CC genotype were rare and possibly originated from loss-of-heterozygosity at the *ACE2* locus. This could reflect a more recent adaptation to a specific niche or environment [58,59]. The results presented in this manuscript suggest that a new, *C*. *albicans-*specific function for Ace2 during hyphal growth, namely inhibition of cell separation, has been selected in certain lineages, possibly fine-tuning the persistence of multicellular structures in the host.

## Materials and Methods

### Strains and growth conditions

The strains used in this work are listed in Tables 2 and 3. Cells were grown in YPD or in synthetic minimal (SC) medium at 28°C. Hypha formation was induced by supplementing the media with 10% Fetal Calf Serum (FCS) at 37°C. All transformants were checked for correct genome integration by PCR. Construction of strains carrying disruption of different genes was done using the PCR-mediated procedure described previously, using different selectable markers [60–62]. The generation of C-terminal fusions to different fluorescent proteins was performed as previously described [60]. To tag *CDC10* with GFP in the WO-1 strain, plasmid pFA-GFP-SAT1 was used as template.

**Table 2:** Strains used in this study.

Strain	Genotype	Origin
BWP17	*ura3*::*imm434/ura3*::*imm434 his1*::*hisG/his1*::*hisG arg4*::*hisG/arg4*::*hisG*	[63]
BWP17-SHA	*SEP7/SEP7-HA-URA3*	[33]
CAG39	*SEP7/SEP7-GFP-ARG4*	[33]
JC305	*CBK1/CBK1-myc-HIS1*	[64]
JC1165	*mob2-4A-HA-URA3 /mob2*Δ::*ARG4 CDC10/CDC10-GFP-HIS1*	This study
OL1111	*SAT1*-*P*_*MET3*_ *-ace2* ^*S*^ *-HA-URA3/ace2*Δ::*ARG4*	This study
OL1170	*ace2* ^*S*^-*HIS1/ace2*Δ::*ARG4 ace2* ^*L*^ *-URA3*@*RPS1*	This study
OL1203	*SAT1-P*_*MET3*_ *-ace2* ^*L*^ *-HA-URA3/ace2*Δ::*ARG4*	This study
OL1444	*ace2*Δ::*URA3/ace2*Δ::*HIS1 CDC10/CDC10-GFP-ARG4*	This study
OL1451	*ace2*Δ::*HIS1/ace2*Δ::*ARG4*	This study
OL1453	*ace2*Δ::*HIS1/ace2*Δ::*ARG4 SEP7/SEP7-HA-URA3*	This study
OL1457	*ace2*Δ::*HIS1/ace2*Δ::*URA3 SEP7/SEP7-GFP-ARG4*	This study
OL1538	*ACE2-HA-URA3/ace2*Δ::*ARG4*	This study
OL1551	*ace2-*Δ*Zn-HA-URA3/ace2*Δ::*ARG4 CDC10/CDC10-GFP-HIS1*	This study
OL1597	*ace2* ^*L*^ *-HIS1/ace2*Δ::*ARG4*	This study
OL1598	*ace2* ^*S*^ *-HIS1/ace2*Δ::*ARG4*	This study
OL1631	*ace2* ^*L*^ *-HIS1/ace2*Δ::*ARG4 SEP7/SEP7-GFP-URA3*	This study
OL1634	*ace2* ^*S*^ *-HIS1/ace2*Δ::*ARG4 SEP7/SEP7-GFP-URA3*	This study
OL1659	*ace2* ^*S*^ *-HIS1/ace2*Δ::*ARG4 CDC10/CDC10-GFP-URA3*	This study
OL1662	*ace2* ^*L*^ *-HIS1/ace2*Δ::*ARG4 CDC10/CDC10-GFP-URA3*	This study
OL1714	*ace2* ^*S*^ *-HIS1/ace2*Δ::*ARG4 P*_*MET3*_ *-Ace2* ^*L*^ *-A-HA-URA3@RPS1*	This study
OL1716	*ace2* ^*S*^ *-HIS1/ace2*Δ::*ARG4 P*_*MET3*_ *-Ace2* ^*L*^ *-AB-HA-URA3@RPS1*	This study
OL1719	*ACE2-6His-HA-HIS1/ACE2-6His-HA-ARG4 CBK1/CBK1*-*myc*-*URA3*	This study
OL1723	*SAT1*-*P*_*MET3*_ *-ace2* ^*S*^ *-HA-URA3/ace2*Δ::*ARG4 CBK1/CBK1*-*myc*-*URA3*	This study
OL1874	*ace2* ^*S*^ *-HIS1/ace2*Δ::*ARG4 P*_*MET3*_ *-Ace2* ^*L*^ *-ABCD-HA-URA3@RPS1*	This study
OL1876	*ace2* ^*S*^ *-HIS1/ace2*Δ::*ARG4 P*_*MET3*_ *-Ace2* ^*L*^ *-*Δ*B-HA-URA3@RPS1*	This study
OL1890	*ace2* ^*S*^ *-HIS1/ace2*Δ::*ARG4 SEP7/SEP7-HA-URA3*	This study
OL1992	*ace2* ^*L*^ *-HIS1/ace2*Δ::*ARG4 SEP7/SEP7-HA-URA3*	This study
OL1994	*SAT1-P*_*MET3*_ *-ace2* ^*L*^ *-HA-URA3/ace2*Δ::*ARG4 CBK1/CBK1*-*myc*-*URA3*	This study
OL2035	*ace2*Δ::*ARG4/kahu-ACE2*	This study
OL2037	*ACE2/kahu-ace2*Δ::*ARG4*	This study
OL2086	*CDC10/CDC10-mCherry-URA3 SEP7/SEP7-GFP-SAT1*	This study
OL2087	*ace2*Δ::*ARG4/ace2*Δ::*HIS1 CDC10/CDC10-mCherry-URA3 SEP7/SEP7*-*GFP*-*SAT1*	This study
OL2088	*ace2* ^*L*^ *-HIS1/ace2*Δ::*ARG4 CDC10/CDC10-mCherry-URA3 SEP7/SEP7*-*GFP*-*SAT1*	This study
OL2090	*ace2* ^*S*^ *-HIS1/ace2*Δ::*ARG4 CDC10/CDC10-mCherry-URA3 SEP7/SEP7*-*GFP*-*SAT1*	This study
OL2182	*sep7*Δ::*HIS1/sep7*Δ::*SAT1 CDC10/CDC10-GFP-ARG4*	This study
OL2244	*CDC10/CDC10-GFP*	This study
OL2327	*ace2*Δ::*ARG4/SAT1- P*_*MET3*_ *-ace2* ^*L*^ *-GFP*::*HIS NOP1/NOP1*-*mCherry*-*URA3*	This study
OL2345	*ACE2-GFP-HIS/ACE2 NOP1/NOP1-mCherry-URA3*	This study
OL2347	*NOP1/NOP1-mCherry-URA3*	This study
OL2356	*ace2*Δ::*ARG4/SAT1- P*_*MET3*_ *-ace2* ^*S*^ *-GFP*::*HIS NOP1/NOP1*-*mCherry*-*URA3*	This study
WO-1	*MTLalpha*	[40]
WO-1 (TT)	*MTLalpha SAT1-PMET3-ACE2 (TGA25)/ACE2*	This study
WO-1 (CT)	*MTLalpha SAT1-PMET3-ACE2 (CGA25)/ACE2*	This study
WO-1 10G	*MTLalpha CDC10/CDC10-GFP*-*SAT1*	This study

**Table 3:** *C*. *albicans* natural isolates used in this study.

Strain	Alias	Clade	Genotype	Origin
CEC3629	HE077_10	1	TT	Guiana
CEC3682	EGPHCRM2 (182)	1	CT	France
CEC3539	Bougn01	2	TT	France
CEC3626	DPC46	3	CT	Belgium
CEC3631	HE076_10	3	TT	Guiana
CEC3689	EGPURRC2 (194)	3	CT	France
CEC3530	C1_X	4	TT	France

### Plasmid constructions

Plasmid pFA-GFP-SAT1 was constructed by cloning an 885 bp *Hin*dIII fragment containing GFP obtained from pFA-GFP-CaURA3 [60] into the *Hin*dIII site of pFA-SAT1 [61]. Plasmids pC1158 and pC1159, containing the *ACE2* promoter and point mutations at the first or second methionine respectively, were constructed in two steps. First, a DNA fragment from the 5′ region of *ACE2* (-1281 to -884) was PCR amplified with specific oligonucleotides that generated *Bam*HI and *Spe*I sites at the ends and the amplified fragment was cloned into the same sites of vector pFA-CaHIS1-MET3p [60]. Then, the *ACE2* promoter (from -857 to -1) and the first amino acids of the coding sequence were amplified with oligonucleotides that introduced point mutations at the first or second ATG and generated the *Pme*I and *Spe*I sites. These fragments were cloned into the same sites of the previous plasmid, replacing the *MET3* promoter with *ACE2* sequences to yield pC1158 and pC1159. The presence of the mutations was confirmed by sequence analysis.

To construct the plasmids carrying different regions of *ACE2* under the control of the *MET3* promoter used to integrate them at the *RPS1* locus, different regions of the gene were amplified using specific oligonucleotides that generated *Bam*HI and *Spe*I sites at the ends and the different fragments were cloned at the same sites of the pCaEXP-HA vector. This generated fusions of the last amino acid of each amplified fragment to the HA epitope. Using this approach, plasmids pC1257 (carrying *ACE2* ^*L*^, nucleotides 1–2349), pC1229 (*ACE2* ^*L*^-ABCD, 1–1923), pC1231 (*ACE2* ^*L*^-AB, 1–1005) and pC1225 (*ACE2* ^*L*^-A, 1–672) were constructed. Plasmids pC1230, carrying *ACE2* ^*L*^-ΔB (1–672, 1006–2349), and pC1208, containing *ACE2* ^*L*^-ΔZn (1–1950, 2092–2349) were constructed in a similar way, generating the deletion of the domains by recombinant PCR. The different plasmids were digested with *Stu*I to direct the integration of the linear fragment at the *RPS1* locus. Plasmid pCaEXP-HA is a derivative of pCaEXP [44] that was constructed by PCR amplification of the HA epitope with oligonucleotides that generated *Bam*HI-*Spe*I sites at the 5′ end and *Pst*I at the 3′ end, cloning the fragment in the corresponding sites of the pCaEXP vector.

### Microscopy

Fluorescence microscopy was performed with a Personal Deltavision microscope running softWoRx (Applied Precision Instruments) equipped with a Photometrics CoolSNAP HQ camera. Z-stack images were collected with step sizes of 0.3 μm and were deconvolved using softWoRx. Images are projections of the deconvoluted Z-stacks. Ace2-GFP fluorescence was analyzed using a Olympus IX81 microscope equipped with a spinning-disc confocal system (Roper Scientific) and images are a single focal plane.

For FRAP analysis, cells were grown as hyphae for 1.5–2 hours, mounted on glass slides, and analyzed with a Olympus IX81 microscope equipped with a spinning-disc confocal system and iLas FRAP module (Roper Scientific). Rings were photobleached and pictures were taken every 10 seconds for the first 80 seconds and then every 20 seconds for another 280 seconds to analyze Cdc10 dynamics. The fluorescence intensity of the bleached areas was quantified with ImageJ (http://rsb.info.nih.gov/ij/) and normalized by subtraction of the background and correction for fluorescence loss using non-bleached control rings in the same image series.

### RNA purification and quantitative RT-PCR

To determine the transcription level of genes, cells of the yeast cultures were collected by centrifuging and total RNA was isolated using the TRIZOL method (Invitrogen), according to the manufacturer’s instruction. cDNA synthesis was carried out with the SuperScript II First-Strand Synthesis System (Invitrogen), using 3 μg RNA as template previously treated with DNAase I (Invitrogen). For quantitative PCR (Applied Biosystems 7300 Real-Time PCR System), the SYBR *Premix Ex Taq* (TaKaRa) reagent was used at final primer concentration of 0.2 μM. Serial dilutions of wild-type *C*. *albicans* DNA (1/10, 1/100, 1/1000, 1/10,000, 1/100,000) were prepared to generate a standard curve for each reaction. The reaction conditions were as follows: 95°C for 45 s and 40 cycles of 95°C for 5 s and 60°C for 31 s, followed by a dissociation step at 95°C for 15 s, 60°C for 1 min and 95°C for 15 s. All PCR reactions were normalized to *ADE2* transcription data. The experiments were repeated at least twice using cDNA from different biological repeats.

### Protein extracts, immunoprecipitation and western blot

Protein extracts were prepared from 1.6x10^8^ cells resuspended in 200 μl of lysis buffer (HEPES 50 mM, 70 mM Potassium acetate, 5 mM Magnesium acetate, 1% Triton X-100, 10% glycerol) containing EDTA-free protease inhibitor mix (Roche), and 300 μl of glass beads (0.4 mm; Sigma-Aldrich) were added. Cells were broken for 60 sec in a FastPrep FR120 (Savant, Bio101) and the extract was recovered by washing twice with 400 μl of lysis buffer. Soluble proteins were obtained by centrifuging the extracts at 5000 g for 10 min at 4°C. For immunoprecipitation, protein extracts were immunoprecipitated using anti-HA or anti-myc μMACS Epitope Tag Protein Isolation kits (Miltenyi Biotec) according to the manufacturer’s instructions. For Western blotting, 50 μg of protein extracts were separated by 8–10% SDS-PAGE, transferred to Hybond-P (Amersham Bioscience) membranes, and probed with anti-myc (9E10, 1:5000), anti-HA (3F10 Roche, 1:20000), anti-Cdc11 (Santa Cruz Biotechnology, 1:5000) or anti-PSTAIRE (Sigma-Aldrich, 1:5000) antibodies.

For septin immunoprecipitation, cells were broken in lysis buffer (50 mM Tris-HCl, pH 7.8, 250 mM Potassium acetate, 2 mM MgCl_2_, 0.5 mM EGTA, 0.8% Triton X-100) containing EDTA-free protease inhibitor mix (Roche). 1 mg of protein extracts was used for immunoprecipitation using RFP-Trap (Chromotek). The beads were washed with lysis buffer containing 500 mM (3 times) or 1M potassium acetate (3 times) before elution. Membranes were probed with anti-GFP (Living Colors Monoclonal antibody JL-8, Clontech), Living Colors DsRed polyclonal antibody (Clontech) and anti-Cdc11 (Santa Cruz Biotechnology).

## Supporting Information

S1 Fig Additional phenotypes of *ace2* ^*L*^ */ace2*Δ and *ace2* ^*S*^ */ace2*Δ strains. **A)** Nucleotide changes introduced to generate strains *ace2* ^*S*^ */ace2*Δ and *ace2* ^*L*^ */ace2*Δ. The A of ATG2 was considered as +1. **B)** Differential interference contrast (DIC) images from strains *ACE2 SEP7-GFP* (CAG39), *ace2*Δ/Δ *SEP7-GFP* (OL1457), *ace2* ^*S*^ */ace2*Δ *SEP7-GFP* (OL1634) and *ace2* ^*L*^ */ace2*Δ *SEP7-GFP* (OL1631) during yeast growth. Scale bar, 5 μm. **C)** Morphology of strains *ACE2* (BWP17), *ace2*Δ/Δ (OL1451), *ace2* ^*L*^ */ace2*Δ (OL1631) and *ace2* ^*S*^ */ace2*Δ (OL1634) incubated for 3 hours under inducing conditions. Hyphae were stained with calcofluor white. Images are the maximum projection of 10 z-planes acquired every 0.4 μm. Scale bar, 5 μm. **D)** Examples of separated hyphal bodies in which the first septum was cleaved (1), isolated hyphae without a cell body (2) or hyphae in which the first septum was being degraded (3) in strain *ace2* ^*S*^ */ace2*Δ.
(TIF)

S2 Fig Characteristics of *C. albicans* and *S. cerevisiae* Ace2 A) Nucleotide sequence of the *ACE2* region around ATG1 in BWP17 and WO-1 strains. A single nucleotide polymorphism (SNP) is present in the ninth codon of the coding sequence of the long form of *ACE2*. The CGA codon that codes for Arg in BWP17 cells (CC genotype) is replaced by a TGA stop codon in both alleles (TT genotype). **B)** Comparison of Ace2 from *S*. *cerevisiae* and *C*. *albicans*. Schematic representation of the regions identified in *Sc*Ace2 and their possible correspondence with *Ca*Ace2. The percentage of similarity between each region is indicated. **C)** Western blot analysis of strains *P*_*MET3*_ *-ace2* ^*L*^ *-HA* (OL1203), *P*_*MET3*_ *-ace2* ^*S*^ *-HA* (OL1111), *ace2* ^*S*^ */ace2*Δ *ace2* ^*L*^::*RPS1* (OL1170), *ace2* ^*S*^ */ace2*Δ *ace2* ^*L*^ *-ABCD*::*RPS1* (OL1874), *ace2* ^*S*^ */ace2*Δ *ace2* ^*L*^ *-AB*::*RPS1* (OL1716), *ace2* ^*S*^ */ace2*Δ *ace2* ^*L*^ *-A*::*RPS1* (OL1714) and *ace2* ^*S*^ */ace2*Δ *ace2* ^*L*^ *-*Δ*B*::*RPS1* (OL1876).
(TIF)
