# Supplementary figures and images for: A Single Nucleotide Polymorphism Uncovers a Novel Function for the Transcription Factor Ace2 during Candida albicans Hyphal Development
Source: PLoS Genet. 2015 Apr 15;11(4):e1005152. doi: 10.1371/journal.pgen.1005152 (PMC4398349; doi:10.1371/journal.pgen.1005152)

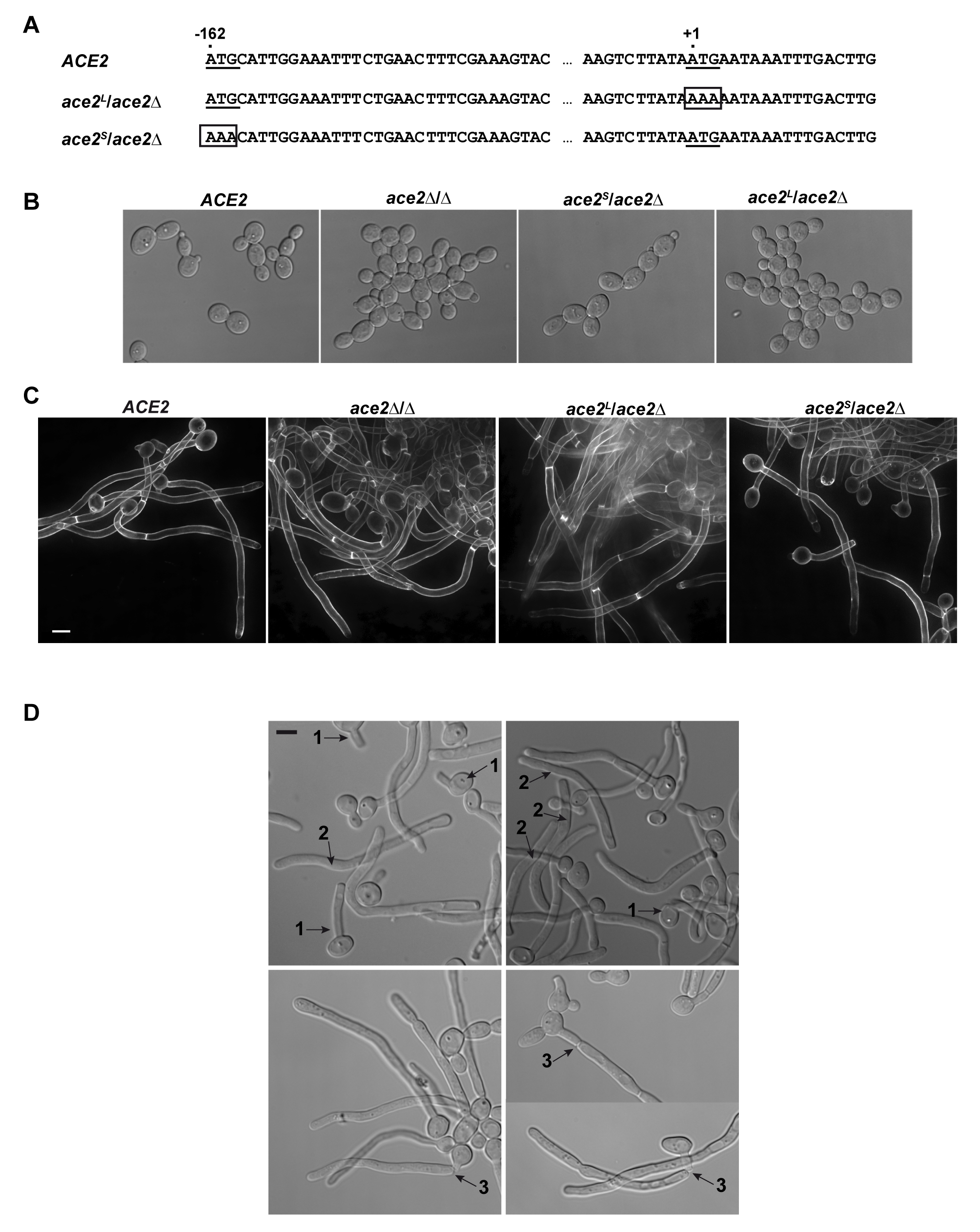

Supplement: S1 Fig — A) Nucleotide changes introduced to generate strains ace2 S /ace2Δ and ace2 L /ace2Δ. The A of ATG2 was considered as +1. B) Differential interference contrast (DIC) images from strains ACE2 SEP7-GFP (CAG39), ace2Δ/Δ SEP7-GFP (OL1457), ace2 S /ace2Δ SEP7-GFP (OL1634) and ace2 L /ace2Δ SEP7-GFP (OL1631) during yeast growth. Scale bar, 5 μm. C) Morphology of strains ACE2 (BWP17), ace2Δ/Δ (OL1451), ace2 L /ace2Δ (OL1631) and ace2 S /ace2Δ (OL1634) incubated for 3 hours under inducing conditions. Hyphae were stained with calcofluor white. Images are the maximum projection of 10 z-planes acquired every 0.4 μm. Scale bar, 5 μm. D) Examples of separated hyphal bodies in which the first septum was cleaved (1), isolated hyphae without a cell body (2) or hyphae in which the first septum was being degraded (3) in strain ace2 S /ace2Δ. (TIF) [file pgen.1005152.s001.tif]

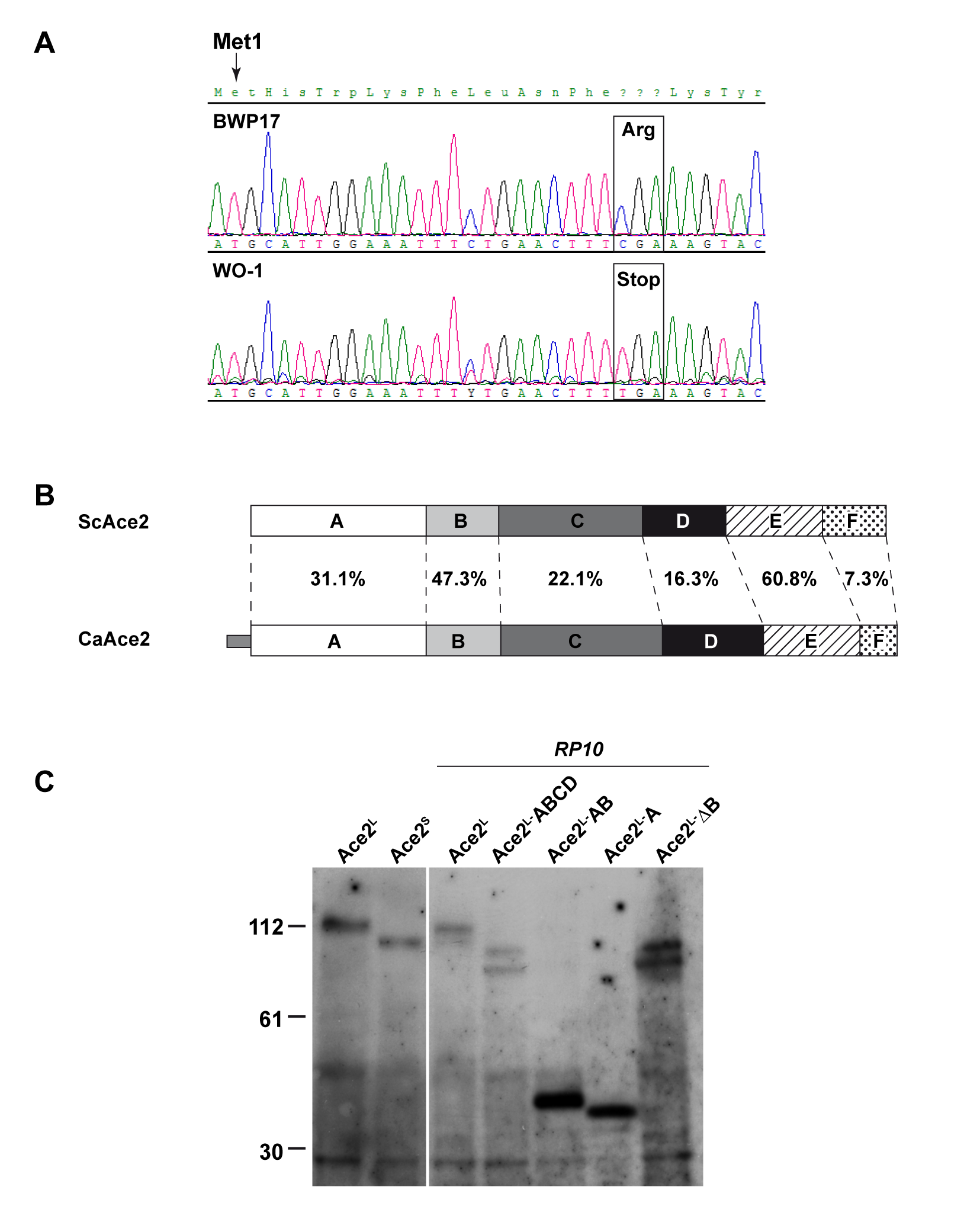

Supplement: S2 Fig — A) Nucleotide sequence of the ACE2 region around ATG1 in BWP17 and WO-1 strains. A single nucleotide polymorphism (SNP) is present in the ninth codon of the coding sequence of the long form of ACE2. The CGA codon that codes for Arg in BWP17 cells (CC genotype) is replaced by a TGA stop codon in both alleles (TT genotype). B) Comparison of Ace2 from S. cerevisiae and C. albicans. Schematic representation of the regions identified in ScAce2 and their possible correspondence with CaAce2. The percentage of similarity between each region is indicated. C) Western blot analysis of strains P MET3 -ace2 L -HA (OL1203), P MET3 -ace2 S -HA (OL1111), ace2 S /ace2Δ ace2 L::RPS1 (OL1170), ace2 S /ace2Δ ace2 L -ABCD::RPS1 (OL1874), ace2 S /ace2Δ ace2 L -AB::RPS1 (OL1716), ace2 S /ace2Δ ace2 L -A::RPS1 (OL1714) and ace2 S /ace2Δ ace2 L -ΔB::RPS1 (OL1876). (TIF) [file pgen.1005152.s002.tif]
